# Beyond quantum linear optics with adaptive boson sampling

**DOI:** 10.1038/s41566-026-01959-3

**Published:** 2026-07-20

**Authors:** Giovanni Rodari, Tommaso Francalanci, Eugenio Caruccio, Francesco Hoch, Giorgio Milani, Gonzalo Carvacho, Taira Giordani, Nicolò Spagnolo, Riccardo Albiero, Niki Di Giano, Giacomo Corrielli, Andrea Crespi, Francesco Ceccarelli, Mattia Bossi, Abhiram Rajan, Roberto Osellame, Ulysse Chabaud, Fabio Sciarrino

**Affiliations:** 1 Dipartimento di Fisica, Sapienza Università di Roma, Rome, Italy; 2 Istituto di Fotonica e Nanotecnologie, Consiglio Nazionale delle Ricerche (IFN-CNR), Milan, Italy; 3 Dipartimento di Fisica, Politecnico di Milano, Milan, Italy; 4 DIENS, École Normale Supérieure, PSL University, CNRS, Paris, France

**Keywords:** Single photons and quantum effects, Quantum optics, Quantum information

## Abstract

Universal photon-based quantum computing requires optical nonlinearities, which can be induced by intermediate measurements and by adaptivity, to be supplied to linear-optical elements. In a near-term perspective, it is essential to probe whether dynamics going beyond linear optics can be accessed with a limited amount of resources. Although recent results show how linear-optical dynamics implies bounds on the set of photonic states that can be generated, quantitative methods for studying the emergence of nonlinear dynamics are largely missing. Here we analyse a regime in which such bounds can be surpassed. We do this by leveraging an adaptive boson sampling architecture in which the optical evolution implemented in a photonic device is progressively adapted via measurement-based feedback. We introduce practical methods to quantify the emergence of a gap with respect to linear optics and derive nonlinearity witnesses from the properties of linear-optical evolution. Then, we validate the toolbox developed within adaptive boson sampling architectures of increasing complexity implemented on a state-of-the-art photonic platform, both by realizing real-time adaptivity and by emulating adaptive protocols via post-selection for more complex configurations. In particular, we probe experimentally a regime in which nonlinear dynamics, unobtainable within a linear-optical paradigm, can arise, thus showing how optical architectures with limited adaptivity are a powerful testbed for exploring new regimes.

## Abstract (web summary)

An adaptive boson sampling architecture is developed to achieve optical evolutions beyond the usual quantum linear optics set-ups via measurement-based feedback. A set of witnesses to certify the nonlinear-optical evolution is provided.

## Main

Although current photonic technologies^1–3^ still require a technological leap to reach the functionalities of a fully fledged universal photonic quantum computer, several instances of architectures tailored to realize non-universal models of photon-based computation, based on the boson sampling (BS) paradigm and its variants^4–10^, have been recently implemented^11–17^. However, the dynamics underlying the standard BS proposal—obtained with solely linear-optical elements—while still being hard to simulate with classical hardware, is known to be a restricted model for photonic quantum computation^5^. To realize a fully universal scheme, it is necessary to introduce a sufficient number of effective optical nonlinearities^18^. Recent studies have investigated how to introduce such effects, either directly via effective interaction dynamics within nonlinear media^19–23^ or by supplying other ancillary resources together with measurement-based adaptivity of the optical evolution^24–27^. The former approach is technologically challenging to achieve^18^, whereas the latter introduces an overhead in terms of the number of optical components^24^ or of the dimensionality of the required photonic resources^26,27^.

Between linear-only BS evolution and universal schemes based on a sufficient number of nonlinearities, which can be understood as the extreme scenarios for photonic quantum computing, one can define a range of intermediate cases where only a moderate number of nonlinearities is present. Specifically, research efforts^10,14,28–30^ have focused on whether the BS paradigm could be progressively extended by adding specific elements and functionalities. Indeed, the BS model integrated with a sufficient number of nonlinear features does retrieve universality^24^. The so-called adaptive boson sampling (ABS) paradigm was recently developed as such an intermediate regime^9^. The ABS architecture uses an adaptive optical interferometer. Specifically, a given number of intermediate measurements are carried out and, according to the measured outcome, the subsequent optical evolution is adaptively reconfigured. The protocol was first implemented on a photonic platform in ref. ^17^, where adaptivity was emulated via post-selection.

Given such partial adaptivity and the presence of coalescence effects with ancillary photons, one might ask whether in such a regime one can access a dynamical evolution that goes beyond linear optics. Are there ABS output photonic states that cannot be prepared deterministically in an equivalent linear-optical picture? How can we characterize this new regime?

Specific tools must be developed to probe the emergence of such a gap. Some previous results in this direction have been reported, largely focused on giving bounds on the set of accessible linear-optical evolutions in Fock space using group-theoretic and geometric methods^31–36^ or in a continuous-variable paradigm^37–39^.

In this work, we develop specific tools to probe nonlinear dynamics, employing them in an experimental setting to show that via ABS one can access a regime that, indeed, goes beyond linear optics. A set of witnesses for the emergence of optical nonlinear dynamics are introduced. These witnesses are based on the measurement of the output Fock-basis density matrix, and they use quantities associated with the Lie algebraic structure at the core of a linear-optical framework^34,40^. Furthermore, such witnesses are tested in experiments of increasing complexity by employing a hybrid photonic architecture tailored for multi-photon experiments^17,40–43^. Adaptivity is implemented through real-time feed-forward, such that the optical circuit is actively reconfigured conditioned on measurement outcomes, for an ABS configuration with two output photons and two output modes. For more complex experiments with up to *n* = 4 input photons and increasing numbers of output photons and modes and involving two different photonic integrated devices, the ABS protocol is emulated via post-selection. Although this approach requires sampling over several fixed interferometric configurations and requires the data to be recombined to emulate a single ABS experiment, post-selection provides an experimentally accessible route for emulating adaptive protocols in increasingly complex regimes.

Overall, our results show how the ABS paradigm enables one to access resources that are not obtainable when relying on only linear-optical dynamics, namely the dynamics implied by a BS architecture.

## The ABS paradigm

The general idea underlying the ABS protocol, as introduced in ref. ^9^, is described below. Consider an optical interferometer with *m* spatial modes and an *n*-photon state $| {\bf{s}}\rangle =| {1}^{\otimes n}{0}^{\otimes (m-n)}\rangle$ at the input. In the general case, the ABS paradigm can involve a cascade of adaptive stages in which several intermediate measurements are performed sequentially, and each measurement outcome conditions a subsequent adaptive unitary acting on the remaining modes.

Overall, an adaptive interferometer ${\mathcal{U}}$ associated with a given ABS scheme can be understood as a set of non-adaptive interferometers described by a unitary matrix *U*_**p**_, each associated with an adaptive outcome string **p** = (**p**^(**1**)^, …, **p**^(**L**)^), where the outcomes ${{\bf{p}}}^{({\bf{l}})}=({p}_{1},\ldots ,{p}_{{k}_{l}})$ are obtained from the measurement of *r*_*l*_ photons in *k*_*l*_ modes at each adaptive stage:

1 $$\left\{\begin{array}{l}{\mathscr{ \mathcal U }}:=\{{U}_{{\bf{p}}},| {\bf{p}}\rangle \in | {{\boldsymbol{\phi }}}_{k,r}\rangle \},\\ {U}_{{\bf{p}}}:=[{{\bf{1}}}_{k}\oplus {V}^{(L)}({{\bf{p}}}^{({\bf{L}})})]\cdots [{{\bf{1}}}_{{k}_{1}}\oplus {V}^{(1)}({{\bf{p}}}^{({\bf{1}})})]\cdot {U}_{0},\end{array}\right.$$

where $k={\sum }_{l=1}^{L}{k}_{l}$ and $r={\sum }_{l=1}^{L}{r}_{l}$, {*V*^(*l*)^(**p**^(**l**)^)} denotes a sequence of adaptive unitaries, each conditioned on the corresponding intermediate measurement outcome **p**^(**l**)^, and **1**_*k*_ denotes the identity matrix on *k* modes.

Conditioned on the observation of a given adaptive measurement outcome, the output state encoded by a superposition of Fock-basis states of ${n}^{{\prime} }=n-r$ photons in ${m}^{{\prime} }=m-k$ modes is given by:

2 $$|{\psi }_{{\bf{p}}}\rangle \langle {\psi }_{{\bf{p}}}|=\frac{1}{N}{\mathrm{Tr}}_{k}[(|{\bf{p}}\rangle \langle {\bf{p}}|\otimes {\hat{{\bf{1}}}}_{m-k}){\hat{U}}_{{\bf{p}}}|{\bf{s}}\rangle \langle {\bf{s}}|{\hat{U}}_{{\bf{p}}}^{\dagger }],$$

where the hat notation (for example, $\widehat{O}$) is used to denote quantum operators acting on the Fock space.

**Fig. 1:**
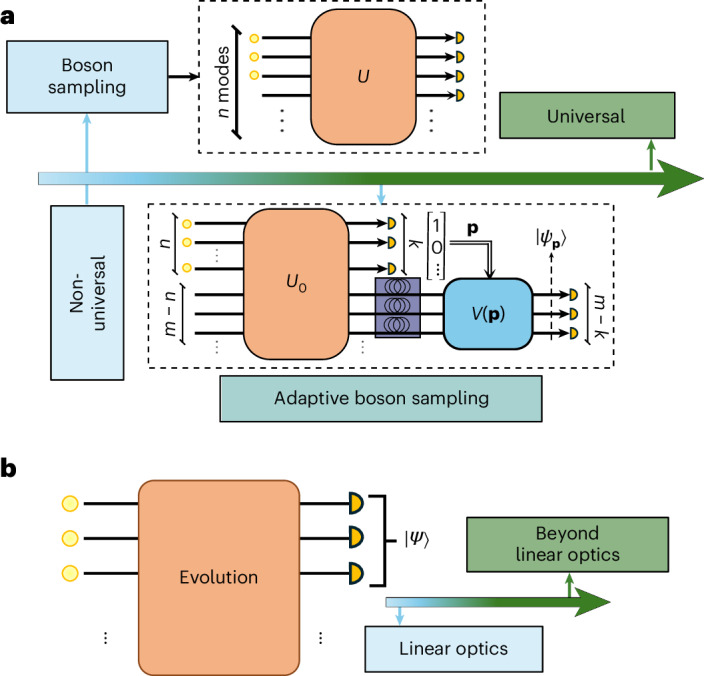
Conceptual scheme of ABS and its verification. **a**, The BS paradigm uses a passive linear-optical dynamics to implement non-universal models for photonic computation. By supplying a sufficient amount of ancillary resources and measurement feed-forward, universal functionalities can be achieved. Between these two extremal scenarios for photonic quantum computing, the ABS paradigm was developed as an extension of BS in which a finite amount of measurement-induced adaptivity is introduced. In the single-stage ABS architecture considered here, *n* denotes the number of input photons, *m* the total number of modes and *k* the number of modes dedicated to adaptive measurements. The interferometer is constructed as a sequence of a unitary *U*_0_ acting on all *m* modes followed by a unitary *V*(**p**) acting on the last *m* − *k* modes, which depends on the measurement outcome **p** for the photons detected in the *k* adaptive measurement modes. **b**, To probe the dynamical evolution going beyond linear optics, a set of measurement-informed quantifiers on the output state $| \varPsi \rangle$ of an optical network is introduced. Such quantifiers are employed as witnesses for the emergence of nonlinear dynamics.

Unless otherwise specified, in the following sections we focus on a single adaptive unitary, which is shown schematically in Fig. 1a, as it captures the essential features of the ABS paradigm. Extended configurations involving several adaptive stages are discussed explicitly when relevant.

Reference ^9^ provides a complete analysis of the classical simulability of the ABS protocol and how it can be employed as a primitive for typical quantum machine-learning architectures^44^. Moreover, a first experimental implementation of the ABS protocol on a photonic platform, in which adaptivity was emulated via post-selection, was performed in ref. ^17^.

## Witnessing nonlinear dynamics

By focusing on the output resources generated by an ABS architecture, the overall goal of this work is to analyse whether a regime going beyond linear optics can be accessed in a scenario with limited adaptivity (Fig. 1b). Specifically, we focus on scenarios involving more than one output photon $({n}^{{\prime} } > 1)$ traversing the ${m}^{{\prime} }$ output modes, in contrast to our previous implementation^17^ where ${n}^{{\prime} }=1$. Indeed, ${n}^{{\prime} } > 1$ is a minimal requirement to access nonlinear dynamics, as it has been shown that all possible states of a single photon in *m* modes are obtainable from a given input using linear optics only^32^ by providing a suitably constructed and sufficiently deep ${m}^{{\prime} }$ mode linear-optical network. Conversely, when considering ${n}^{{\prime} } > 1$, it has been proven that due to arguments related to the physics of linearly interacting bosonic systems, some constraints do arise that effectively reduce the set of accessible output states from a given Fock state input^31–33^. Therefore, within an ABS protocol with ${n}^{{\prime} } > 1$ photons at the output and where a degree of measurement-induced nonlinearity is present, it may be possible to observe a dynamical evolution that bypasses these constraints with respect to an equivalent linear-optical model, thus allowing an ABS architecture to generate at its output photonic resources that would otherwise be inaccessible.

### Passive separability and emergence of optical nonlinearities

To verify the generation of resources inaccessible in the linear regime, one needs to define suitable and measurable witnesses attesting to the emergence of dynamics going beyond linear optics. As described below, we employ two parallel approaches, which can both be related to the general concept of passive separability introduced in ref. ^38^. Namely, a state $| \phi \rangle$ is passively separable if there exists a passive linear unitary operator $\widehat{V}$ such that $\widehat{V}| \phi \rangle$ is separable^45^. The role of this concept in photonic quantum computational advantage was analysed in ref. ^46^, which also showed how the lack of passive separability implies the emergence of a so-called non-Gaussian entangled resource that has a direct impact on bosonic-based quantum computation. Notably, this means that states lacking passive separability are entangled in any mode basis. Crucially, here we show explicitly that passively separable states that are eigenstates of the number operator have a specific structure:

**Theorem 1**. *Let* $\widehat{N}={\sum }_{i=1}^{m}{\widehat{a}}_{i}^{\dagger }{\widehat{a}}_{i}$ *be the number operator over* *m* ≥ 0 *modes. Let* $| \psi \rangle$ *be a pure state over m modes such that* $\widehat{N}| \psi \rangle =n| \psi \rangle$, *with* *n* ≥ 0. *A Fock-basis state* $| \psi \rangle$ *is passively separable if and only if there exists an* *n*-*photon pure state* ${\otimes }_{i=1}^{m}| {n}_{i}\rangle$, *with* ${\sum }_{i=1}^{m}{n}_{i}=n$, *and an m-mode interferometer* *U* *such that* $| \psi \rangle =\widehat{U}{\otimes }_{i=1}^{m}| {n}_{i}\rangle$.

This new result is proven in ‘Proof of the main theorem about passive separability’ in Methods. It states that the class of passively separable states with a well-determined total number of photons *n* corresponds to the overall set of resources that can be generated at the output of an equivalent passive BS device fed with one from among all the possible Fock-basis states in the form ${\otimes }_{i=1}^{m}| {n}_{i}\rangle$ with ${\sum }_{i=1}^{m}{n}_{i}=n$. In other words, if the initial state at the input of an interferometer is separable across the optical modes, then showing that the output state lacks passive separability means that it can be generated only with an evolution that goes beyond linear optics.

The first approach developed here aims to quantify a distance metric between the set of optical states constrained by a linear-optical evolution and a fixed output state obtained in an ABS configuration. This approach relies on the full reconstruction of its density matrix in a Fock-basis representation. Although some general schemes have been proposed to perform tomography of states in a high-dimensional Fock space^47^, such procedures become increasingly impractical for a high number of photons or modes. Therefore, in this experimental validation, this criterion is applied only in a two-photon, two-mode regime, following the procedure detailed in Supplementary Section 1.

Given knowledge of the output state Fock-basis density matrix ${\widehat{\rho }}_{i}$, one can check for the lack of passive separability by evaluating the distance between ${\widehat{\rho }}_{i}$ and the set of states that can be obtained in an equivalent linear-optical framework. This is equivalent to solving the following problem:

3 $$W(\widehat{\rho })=\mathop{\min }\limits_{{\widehat{{\boldsymbol{\sigma }}}}_{| {n}_{1},{n}_{2}\rangle }}(1-F(\widehat{\rho },\widehat{\sigma }))\ge 0.$$

Here ${\widehat{{\boldsymbol{\sigma }}}}_{| {n}_{1},{n}_{2}\rangle }$ identifies the set of states achievable by a passive BS scheme with an input two-photon state $| {n}_{1},{n}_{2}\rangle$ and an arbitrary two-mode optical evolution. $F(\widehat{\rho },\widehat{\sigma })$ denotes the standard fidelity metric^48^. Overall, if one can show that $W({\widehat{\rho }}_{i}) > 0$ holds with respect to both ${\widehat{{\boldsymbol{\sigma }}}}_{| 1,1\rangle }$ and ${\widehat{{\boldsymbol{\sigma }}}}_{| 2,0\rangle }$, then ${\widehat{\rho }}_{i}$ will lack passive separability, here understood as the emergence of a nonlinear signature associated with the optical evolution implied by the ABS architecture.

### Violation of Lie algebraic invariants as a nonlinearity witness

When a full reconstruction of an output state in the Fock-basis representation becomes unfeasible, one can still probe deviations from linear-optical dynamics. This can be achieved by leveraging Lie algebraic invariants. These invariants were proposed in ref. ^34^ to study the fundamental constraints associated with a linear-optical evolution, specifically in a passive BS paradigm, from the perspective of photonic state preparation. Notably, it was shown how the unitary dynamics implemented by passive linear-optical interferometers can be related to the structure of Lie algebras. As a consequence, numerical quantities that can be computed from suitable measurements on a Fock-basis state are invariant throughout a linear-optical evolution.

Specifically, the following expression can be considered:

4 $$I(\widehat{\rho })=\mathop{\sum }\limits_{i=1}^{{m}^{2}}{({\rm{Tr}}({\widehat{O}}_{i}\widehat{\rho }))}^{2},$$

which depends on the expectation values of a set of *m*^2^ operators ${\widehat{O}}_{i}$, as defined in ‘Lie algebraic observables’ in Methods. It can be proven that under a linear-optical evolution, described by a unitary operator ${\widehat{U}}_{{\rm{lin}}}$, the value attained by equation (4) is invariant:

5 $${\widehat{\rho }}_{{\rm{in}}}\mathop{\longrightarrow }\limits^{{\widehat{U}}_{{\rm{lin}}}}{\widehat{\rho }}_{{\rm{out}}}\,\Rightarrow \,I({\widehat{\rho }}_{{\rm{in}}})=I({\widehat{\rho }}_{{\rm{out}}}),$$

where ${\widehat{\rho }}_{{\rm{in}}}$ identifies a fixed input state for the linear-optical network. The invariance properties defined by equation (5) and the associated linear-optical dynamics were recently tested and experimentally confirmed in a broad range of passive BS experiments^40^.

Conversely, whenever $I({\widehat{\rho }}_{{\rm{in}}})\ne I({\widehat{\rho }}_{{\rm{out}}})$, one can conclude that one of the underlying constraints, namely the assumption of the optical dynamics being linear, is violated. Overall, one can interpret a Lie algebraic invariant not only as a tool to evaluate whether a photon state can be prepared via linear optics but also as a witness of the emergence of nonlinear-optical dynamics. In the present context, the lack of passive separability for a given ${m}^{{\prime} }$-mode, ${n}^{{\prime} }$-photon state $\widehat{\rho }$ at the output of an ABS dynamics can be probed by showing that its associated invariant $I(\widehat{\rho })$ deviates from the set of possible invariants that can be obtained in any passive BS experiment, that is those computed on all ${n}^{{\prime} }$-photon states in the form $| {\mathbf{s}}\rangle ={\otimes }_{j=1}^{{m}^{{\prime} }}| n^{\prime}_{j} \rangle$ with ${n}^{{\prime} }={\sum }_{j=1}^{{m}^{{\prime} }}n^{\prime}_{j}$. Finally, we also note that if we are to draw conclusions about the lack of passive separability of the state $\widehat{\rho }$ at the output of a given optical protocol, a description of the underlying optical evolution generating it is not required.

## Experimental results

**Fig. 2:**
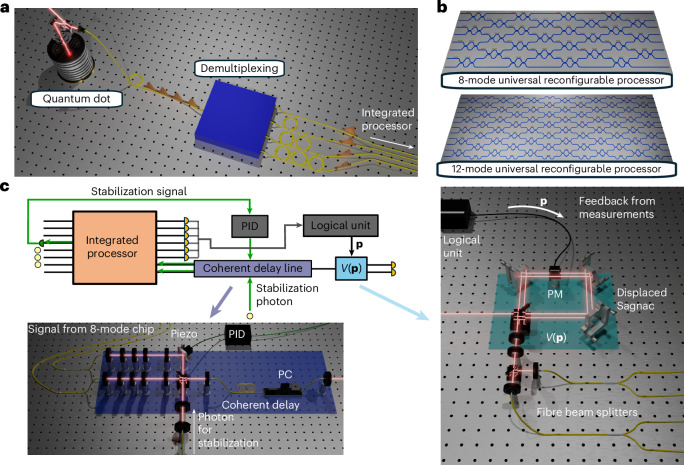
Experimental scheme used to verify the nonlinear behaviour of the ABS scheme via QOLOSSUS-PRO. **a**, Experimental scheme to generate multi-photon input states. A quantum-dot single-photon source is interfaced with a time-to-space demultiplexing stage to generate synchronized multi-photon states with up to four photons distributed over distinct spatial modes. **b**, Experimental layout of the reconfigurable photonic processing stage. The multi-photon states are injected into programmable photonic integrated circuits (PICs) with either 8 or 12 spatial modes, which are configured to implement the ABS interferometric configurations employed in this work. **c**, Experimental scheme for the realization of adaptive measurements and real-time feed-forward operations. A three-photon input state is first processed by an initial unitary *U*_0_ implemented on the eight-mode PIC. Six modes are used in the adaptive measurement of one photon, and the remaining two-mode, two-photon state is routed to the adaptive processing stage. The latter undergoes polarization conversion and active phase stabilization, assisted by an auxiliary stabilization photon counter-propagating through the chip, which provides feedback to a proportional–integral–derivative control loop acting on a piezo-electric element. To enable real-time feed-forward, the polarization-encoded state is delayed with a 240-m-long optical fibre compensated with a polarization controller. The state is converted back to spatial encoding via a displaced Sagnac interferometer. An electro-optic phase modulator applies at this stage a measurement-conditioned phase shift depending on the outcome **p** measured by an avalanche photodiode and processed by a logical unit, effectively implementing the adaptive transformation *V*(**p**). Finally, the output state is analysed using a pseudo-number-resolving detection scheme exploiting fibre beam splitters. PC, polarization controller; PID, proportional–integral–derivative; PM, phase modulator.

To provide an experimental demonstration of the nonlinearity of the resource states produced via the ABS scheme, we employed a state-of-the-art set-up comprising several interconnected stages. The QOLOSSUS-PRO photonic machine has a demultiplexed quantum-dot-based source (Fig. 2a), interfaced with either an 8-mode or a 12-mode programmable integrated interferometer^17,40–43^ (Fig. 2b). For more details of the experimental apparatus, refer to ‘Experimental apparatus’ in Methods.

As mentioned in ‘Witnessing nonlinear dynamics’, within an ABS scheme, the minimal requirement for observing nonlinear dynamics going beyond linear optics is to have ${n}^{{\prime} } > 1$ photons distributed on ${m}^{{\prime} } > 1$ output modes.

To benchmark the experimental platform and validate its ability to implement ABS protocols, we performed two kinds of experiments in this non-trivial regime.

**Fig. 3:**
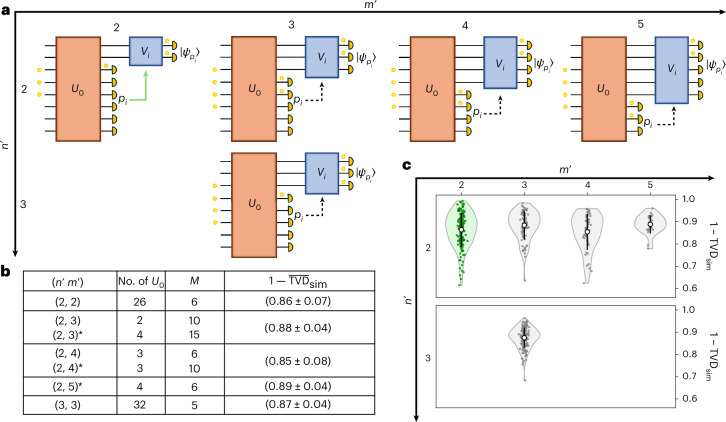
Implementation of ABS experiments with an increasing number of output photons and modes. **a**, Diagrams of the implemented ABS experiments with *m* = 8 modes and increasing numbers of output modes ${m}^{{\prime} }$ and output photons ${n}^{{\prime} }$. In the (2, 2) case, the experiment implements real-time adaptivity, which is represented by the solid green arrow. Data from the real-time adaptive experiment are also highlighted in green in **c**. In all other configurations, adaptivity is emulated via post-selection (dashed arrows). In this case, the experiments have *n* = 4 input photons and detect *r* photons in the adaptive measurement modes, thus resulting in an output state of ${n}^{{\prime} }$ photons in ${m}^{{\prime} }$ modes. **b**, Summary table reporting, for each configuration, the number of sampled input transformations *U*_0_, the number *M* of measurement outcomes and corresponding adaptive unitaries (**p**_*i*_, *V*_*i*_), and the average value of 1 − TVD_sim_, where TVD_sim_ is the total variation distance with respect to the numerical simulations (with a model accounting for experimental imperfections), averaged over all sampled instances of *U*_0_ and *V*_*i*_. Configurations in which bunching events are also considered to be valid adaptive measurement outcomes **p**_*i*_ are marked with an asterisk: ${({n}^{{\prime} },{m}^{{\prime} })}^{* }$. **c**, Violin plots showing the distribution of the values for 1 − TVD_sim_, taken as a metric of the quality of the overall implementation, for the different output configurations $({n}^{{\prime} },{m}^{{\prime} })$. Each data point corresponds to a distinct experimentally sampled adaptive configuration, defined by a sampled input interferometer *U*_0_ and a measurement-conditioned adaptive evolution (**p**_*i*_, *V*_*i*_). For each $({n}^{{\prime} },{m}^{{\prime} })$ configuration, the total sample size is given by the total number of sampled adaptive instances reported in **b**, namely ${N}_{{\rm{tot}}}={\sum }_{x}\#{U}_{0}^{(x)}{M}^{(x)}$, where the sum includes both standard and starred configurations when present. The violin plots report the full distribution of the sampled values. Central markers and error bars indicate mean values ± standard deviations.

The first employs real-time adaptivity and effectively implements a single-stage ABS experiment that probes nonlinearity in the case with ${n}^{{\prime} }=2$ output photons and ${m}^{{\prime} }=2$ output modes. In this experiment, genuine feed-forward control is implemented following the scheme shown in Fig. 2c and described in detail in Supplementary Section 2. We briefly summarize it below. After the intermediate detection of a single photon (*r* = 1) in *k* = 6 modes, the two undetected outputs are converted into polarization-encoded modes through a path-to-polarization converter with active phase stabilization (Fig. 2c). This procedure is crucial for phase-stable propagation outside the photonic chip. Then, the two-photon polarization state is temporally delayed using a 240-m-long optical fibre, which provides sufficient time to process the measurement outcome and apply the corresponding adaptive operation. The photons are then converted back into spatial modes using a polarization-to-path interferometric stage (a displaced Sagnac interferometer), during which an adaptive phase shift is applied in real time to one interferometer arm via a fast electro-optic modulator. The applied phase depends explicitly on the detected measurement outcome, which determines the voltage applied to the electro-optic modulator within a predefined evenly spaced range, thereby realizing an outcome-conditioned unitary transformation *V*(**p**). The corresponding ABS configuration is illustrated in Fig. 3a, in particular in the upper left diagram for $({n}^{{\prime} },{m}^{{\prime} })=(2,2)$. The green arrow indicates the real-time implementation of adaptivity.

The second kind of experiment implements single-stage ABS architectures featuring output states with ${n}^{{\prime} }=\{2,3\}$ photons across ${m}^{{\prime} }=\{3,4,5\}$ modes. In these cases, the experiments are performed by emulating adaptivity through post-selection, which allows the exploration of more complex output configurations within the same single-stage ABS paradigm. In these experiments, the programmable device with *m* = 8 modes is programmed into two blocks, the first implementing a fixed and randomly extracted unitary transformation *U*_0_ and the other an adaptive unitary *V*_*i*_ acting on ${m}^{{\prime} }$ modes. Diagrams of the implemented configurations are given in Fig. 3a. The dashed arrows indicate that adaptivity is emulated via post-selection. Note that, here and thereafter in the post-selection experiments, the interferometer configuration for the adaptive unitary *V*_*i*_ is chosen according to a fixed, deterministic rule, which is not optimized for any specific task but rather serves as a generic prescription to probe the ABS framework. Specifically, the phases defining *V*_*i*_ are chosen according to a fixed mapping between the ordered measurement outcomes **p**_*i*_, with *i* = 1, …, *M*, and a set of phase values, for example, *θ*_*i*_ = *ϕ*_*i*_ = *i*π/(*M* + 1). Post-selection ensures a consistent correspondence between each adaptive measurement outcome and the applied unitary *V*_*i*_.

The full output photon-count distributions are reconstructed in the experiment via a pseudo-number-resolving technique that also detects events where more than one photon ends up in the same mode, following the data analysis procedure described in ref. ^40^. We note that we record only events in which the number of photons detected at the output of the interferometer is equal to the number of photons injected at its input.

To assess the accuracy of the experimental implementation, we use the total variation distance $\mathrm{TV}{{\rm{D}}}_{\mathrm{sim}}=\frac{1}{2}\parallel {\bf{P}}-{{\bf{P}}}_{\mathrm{sim}}{\parallel }_{1}$ computed between **P**, the measured distribution, and **P**_sim_, obtained with a numerical simulation of the experiment that incorporates realistic noise sources affecting the apparatus. Specifically, **P**_sim_ is numerically computed using a model that captures the dominant non-idealities of the experimental platform, namely partial photon distinguishability and multi-photon emission from the source but assuming that both the unitary transformation *U*_0_ and the adaptive evolution *V*_*i*_ are correctly implemented with no errors. Further details of the numerical model employed are provided in Supplementary Section 3. The table in Fig. 3b lists for each $\{{m}^{{\prime} },{n}^{{\prime} }\}$ configuration the number of sampled *U*_0_ instances, the number *M* of different possible adaptive measurement outcomes **p**_*i*_ and adaptive unitaries *V*_*i*_ and also the average values ${\varDelta }_{\mathrm{TVD}}=1-\mathrm{TV}{{\rm{D}}}_{\mathrm{sim}}$. The full distributions of *Δ*_TVD_ across all the implemented ABS instances are shown in Fig. 3c. On average, values of *Δ*_TVD_ in excess of 0.85 are obtained, thus confirming the high accuracy of the implementation and the overall control of the experimental apparatus.

As a subsequent step, the methods previously described to probe the emergence of nonlinear-optical dynamics within the ABS paradigm are applied. For this purpose, we performed new experimental acquisitions.

**Fig. 4:**
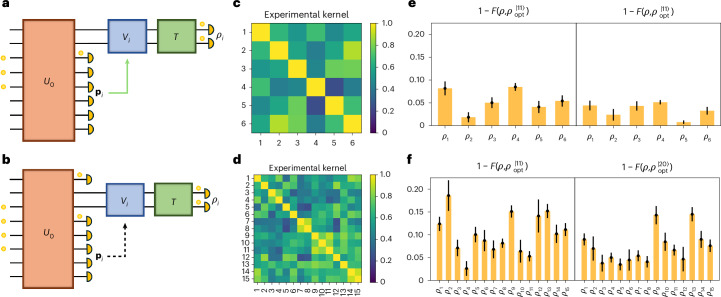
Experimental results for two-photon, two-mode ABS with full output state tomography. **a**,**b**, Diagrams of the experimentally implemented schemes yielding a two-photon output state in two modes, $({n}^{{\prime} },{m}^{{\prime} })=(2,2)$. **a**, Real-time adaptive ABS protocol with *n* = 3 input photons and *r* = 1 measured photon. An initial eight-mode unitary *U*_0_ is followed by an intermediate measurement yielding an outcome **p**_*i*_, which conditions the application of an adaptive two-mode unitary *V*_*i*_ via active feed-forward (solid green arrow). **b**, Post-selected implementation with *n* = 4 input photons and *r* = 2 measured photons. The adaptivity of the operation *V*_*i*_, conditioned on the outcome **p**_*i*_, is emulated in post-selection (dashed arrow). In both cases, the final two-photon output state is characterized via full quantum state tomography, implemented through a set of tomographic projections *T* acting on the output modes. **c**,**d**, Fidelity kernel matrices *K*_*ij*_ computed from the reconstructed output states obtained for a fixed choice of *U*_0_ and different adaptive unitaries *V*_*i*_. **c**, Kernel matrix for the real-time adaptive experiment, constructed from the six reconstructed output states. **d**, Kernel matrix for the post-selected experiment, constructed from the 15 reconstructed output states. **e**,**f**, Minimum distances $1-F(\widehat{\rho },{\widehat{\rho }}^{| 11\rangle })$ and $1-F(\widehat{\rho },{\widehat{\rho }}^{| 20\rangle })$ between the reconstructed output states and the sets of states obtainable via linear-optical evolution from the input states ∣1,1〉 and ∣2,0〉, respectively. **e**, Distances for the six states in the real-time adaptive experiment. **f**, Distances for the 15 post-selected output states. In all cases, the distance is approximated by sampling a representative set of ${\mathcal{O}}(1{0}^{4})$ linear-optical states for each input. Data are presented as mean values ± standard deviation, obtained from Monte Carlo resampling of the experimentally measured tomographic counts assuming Poissonian counting statistics. For each reconstructed output state, the distances were evaluated over *N* = 100 resampled datasets generated from the measured photon-count distributions.

First, we consider two experimental implementations yielding two-photon, two-mode output states $({n}^{{\prime} },{m}^{{\prime} })=(2,2)$, as shown in Fig. 4a,b. The first one is an extension of the real-time adaptive ABS experiment introduced before, with *n* = 3 input photons and *r* = 1 measured photon. The second implementation has *n* = 4 input photons and *r* = 2 measured photons, and in this case, adaptivity is emulated through post-selection. In both implementations, after a fixed evolution *U*_0_, measurement events with *r* = {1, 2} photons are recorded. Each configuration is associated with a different adaptive unitary *V*_*i*_. Then, after the adaptive block, the projective measurements needed to perform a complete tomography of the output state are implemented, as detailed in Supplementary Section 1. For the real-time adaptive experiment, this is performed by exploiting the polarization-encoded representation of the modes, which enables the implementation of the required tomographic projections *T* through a set of wave plates located after the adaptive stage, as described in Supplementary Section 1.

To quantify the accuracy of the reconstruction, the state fidelity between the experimentally reconstructed states ${\widehat{\rho }}_{i}$ and the corresponding states obtained from a numerical simulation that accounts for the dominant noise sources of the experimental apparatus is evaluated. For the real-time adaptive experiment with *n* = 3, we obtained an average fidelity ${\overline{F}}_{n=3}=0.83(6)$. For the post-selected implementation with *n* = 4 input photons, the obtained average fidelity was ${\overline{F}}_{n=4}=0.91(1)$.

The output state distribution in the output Fock space is shown via the pairwise fidelity kernel matrices (Fig. 4c,d), computed as ${K}_{ij}={({\rm{Tr}}\sqrt{\sqrt{{\widehat{\rho }}_{i}}{\widehat{\rho }}_{j}\sqrt{{\widehat{\rho }}_{i}}})}^{2}$. We note that the distribution of ABS output states in Hilbert space can be substantially reshaped by different associations between measurement outcomes and adaptive operations, as discussed in Supplementary Section 4.

Given the knowledge of a given ABS output state, one can numerically evaluate its minimal distance with respect to the set of states obtainable via an equivalent passive linear-optical dynamics, as described in ‘Passive separability and emergence of optical nonlinearities’. To do so, ~10^4^ random states were numerically sampled from the sets of passive separable states ${\widehat{{\boldsymbol{\sigma }}}}_{| 1,1\rangle }$ and ${\widehat{{\boldsymbol{\sigma }}}}_{| 2,0\rangle }$. As shown in Fig. 4e,f, the values obtained for the minimized distances are, in most cases, greater than zero, even when considering the associated experimental errors. Overall, this demonstrates that, even in a small-scale ABS implementation, the observed dynamics can hardly be traced back to equivalent linear BS evolutions. For ${m}^{{\prime} } > 2$, we discuss a similar approach based on the trace distance in Supplementary Section 5, which we apply to the output distributions obtained from post-selection experiments in these regimes.

As a final step, we experimentally implemented ABS schemes with ${m}^{{\prime} }=\{2,3,4\}$ and ${n}^{{\prime} }=\{2,3\}$, focusing on the measurement of the Lie algebraic quantity of equation (5).

**Fig. 5:**
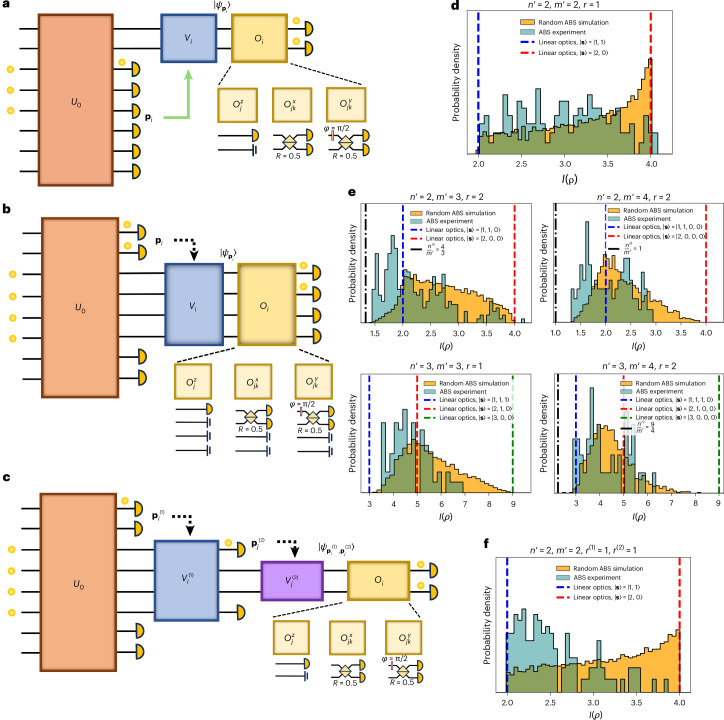
Lie invariants as witnesses of nonlinearity in ABS. **a**–**c**, Conceptual diagrams of the ABS protocols employed to probe Lie invariants. **a**, Real-time adaptive ABS configuration with *n* = 3 input photons and *r* = 1 measured photon, yielding an output state with $({n}^{{\prime} },{m}^{{\prime} })=(2,2)$. An intermediate measurement outcome **p** conditions the application of an adaptive unitary *V* via active feed-forward. The final output state is characterized by directly measuring the expectation values of selected Lie observables. **b**, Single-stage adaptive configuration with *n* = 4 input photons. A measurement outcome **p** conditions a single adaptive unitary *V*, shown here for a representative case with ${m}^{{\prime} }=4$ output modes. **c**, Two-stage adaptive configuration with *n* = 4 input photons. Two sequential measurement outcomes (**p**^(**1**)^, **p**^(**2**)^) condition two adaptive unitaries *V*^(1)^ and *V*^(2)^. In these latter two cases, adaptivity is emulated through post-selection. In all cases, the lower right parts illustrate the linear-optical transformations that need to be implemented to measure the expectation values of specific ${O}_{j}^{z}$, ${O}_{jk}^{x}$ and ${O}_{jk}^{y}$ observables. In the real-time adaptive experiment, these unitaries were implemented using polarization-encoded wave plates, as described in Supplementary Section 1. In the post-selection experiments, they were implemented directly on the integrated photonic chip. **d**–**f**, Experimental results for the measured Lie-invariant quantities $I(\widehat{\rho })$ obtained from ABS experiments. **d**, Measured Lie invariants obtained in the real-time adaptive experiment with *n* = 3 input photons and output configuration $({n}^{{\prime} },{m}^{{\prime} })=(2,2)$. **e**,**f**, Histograms of measured Lie-invariant quantities $I(\widehat{\rho })$ obtained from ABS experiments. In the single-stage configuration (**e**), invariants are reported for different output photon and mode numbers $({n}^{{\prime} },{m}^{{\prime} })=\{(2,3),(2,4),(3,3),(3,4)\}$. In the two-stage adaptive configuration (**f**), invariant values are shown for the probed case $({n}^{{\prime} },{m}^{{\prime} })=(2,2)$. In all cases, the histograms include results from different output states generated by permuting the association of the adaptive unitary to the measurement outcomes. Vertical dashed lines indicate the theoretical values for $I(\widehat{\rho })$ from Fock states with the corresponding number of photons and modes, as well as the theoretical minimum and maximum values allowed in each configuration. We also show results obtained from simulating random ABS output states within the corresponding configuration.

First, we consider again the real-time adaptive set-up introduced above and also extended configurations with up to ${n}^{{\prime} }=3$ output photons and ${m}^{{\prime} }=4$ output modes. The corresponding interferometer diagrams are illustrated in Fig. 5a,b, respectively. For the post-selection regime, we show as a representative case the set-up with ${n}^{{\prime} }=2$ output photons and ${m}^{{\prime} }=4$ output modes. In all cases considered, the final layers of the interferometer were employed to implement the transformations required to measure the observables ${\widehat{O}}_{i}$ used to compute the quantity $I({\widehat{\rho }}_{{\rm{ABS}}})$ for a given output state ${\widehat{\rho }}_{{\rm{ABS}}}$. Specifically, we used a suitable configuration of both balanced and cross beam splitters to couple non-adjacent modes^40^. A schematic representation of the corresponding interferometric architecture is shown in the lower right parts of Fig. 5a,b. In this way, the average number of photons output $\langle {\widehat{n}}_{j}\rangle$ was experimentally measured and used to estimate ${\rm{Tr}}({\widehat{O}}_{i}{\widehat{\rho }}_{{\rm{ABS}}})$. Note that, in general, measuring Lie-invariant observables requires fewer measurements than full state tomography. When ${n}^{{\prime} }={m}^{{\prime} }=2$, the observables required to evaluate $I(\widehat{\rho })$ are a subset of the tomographic measurements, and thus, no further measurements are required in this case.

Figure 5d shows the measured values of $I({\widehat{\rho }}_{{\rm{ABS}}})$ for the real-time adaptive experiments with ${n}^{{\prime} }=2$ and ${m}^{{\prime} }=2$. Figure 5e depicts the distribution of the measured values of $I({\widehat{\rho }}_{{\rm{ABS}}})$ for several emulated ABS configurations defined via the parameters $({n}^{{\prime} },{m}^{{\prime} })$. Note that the post-selection regime in these experiments allows one to consider permutations of the association between measurement outcomes and adaptive unitaries, as explained in Supplementary Section 6, thereby increasing the available statistics. For comparison, the values of $I(\widehat{\rho })$ associated with the relevant passively separable states in each configuration are reported, together with the minimum and maximum value that can be obtained in each configuration. The latter quantities were computed following the procedure reported in ref. ^40^ and are, respectively, $I{(\widehat{\rho })}_{\min }={n}^{{\prime} 2}/{m}^{{\prime} }$ and $I{(\widehat{\rho })}_{\max }={n}^{{\prime} 2}$.

Note that the measured Lie invariant $I({\widehat{\rho }}_{{\rm{ABS}}})$ can substantially deviate from any Lie-invariant value obtainable in an equivalent passive BS dynamics with ${n}^{{\prime} }$ photons in ${m}^{{\prime} }$ modes, which shows that the resources ${\widehat{\rho }}_{{\rm{ABS}}}$ obtained via ABS can be obtained only in a regime going beyond linear optics. We emphasize that the nonlinearity affecting the invariant $I(\widehat{\rho })$ originates from the measurement operation. In fact, different values of $I(\widehat{\rho })$ are observed for different outcomes **p**_*i*_. However, for a fixed measurement outcome **p**_*i*_, the value of $I(\widehat{\rho })$ remains unchanged when permuting the association between **p**_*i*_ and different adaptive unitaries *V*_*i*_. This reflects that, in the single-stage configuration, the conditional evolution induces a linear transformation of the output state. This is discussed in detail in Supplementary Section 6.

All the analysis presented so far has been for a single adaptive stage after the application of the first unitary *U*_0_. Indeed, interesting dynamics arise when several adaptive stages are cascaded. We studied this regime in a set-up where two sequential measurement outcomes (**p**^(**1**)^, **p**^(**2**)^) condition two subsequent adaptive unitaries *V*^(1)^ and *V*^(2)^. This configuration, schematically illustrated in Fig. 5c and described in detail in Supplementary Section 6, results in an output state with $({n}^{{\prime} },{m}^{{\prime} })=(2,2)$, which is again analysed by measuring Lie-invariant observables. Experimentally, this configuration is implemented on a 12-mode integrated photonic processor. We exploited the increased interferometric depth to include several sequential adaptive stages within a single device. Figure 5f shows the experimentally obtained distribution of $I({\widehat{\rho }}_{{\rm{ABS}}})$ in this configuration. In contrast to the single-stage case, the measured invariant values now depend on the choice of the adaptive unitaries, as detailed in Supplementary Section 6. Although *V*^(2)^ acts only as a final linear evolution layer, *V*^(1)^ reshapes the state before the subsequent measurement, thus effectively changing the nonlinear dynamics.

Furthermore, from data obtained in the ${m}^{{\prime} }=\{2,3\}$ scenarios, the spectrum of the state density matrices projected onto the subalgebra of linear-optical Hamiltonians can also be computed. This offers an alternative criterion for showing that the states obtained in the ABS regime are nonlinear, as discussed in Supplementary Section 7.

Finally, note that even if ABS could be employed to access a set of states whose preparation is forbidden with only linear optics, there still exists a regime in which such dynamics is not efficient, for example, when *k* scales linearly with *m*. Notably, when *k* and *r* are allowed to increase, the strategy proposed in ref. ^17^ is to consider mixed states formed by averaging over all (or subsets of) adaptive measurement outcomes. Although the main analysis is focused here on pure states, the mixed-state scenario is investigated in Supplementary Section 8, which confirms that signatures of dynamics beyond linear optics persist in this regime.

## Discussion

In this work, we characterized the quantum resources that can be obtained via the recently proposed ABS paradigm^9^, an intermediate regime towards universal photon-based computation in which a linear-optical apparatus is augmented via measurement-induced adaptivity. In particular, we probed whether the output states of an ABS protocol lack passive separability^46^, meaning that they can be obtained only via beyond linear-optical dynamics. This was validated by introducing practical witnesses for the emergence of such nonlinear behaviours. These witnesses rely either on a full knowledge of the state or on the measurement of the Lie algebraic quantities introduced in refs. ^34,40^. Through the present experimental implementation of ABS schemes of increasing complexity with up to *n* = 4 photons injected into fully programmable integrated photonic processors with either 8 or 12 spatial modes and realized either through real-time adaptivity in simple configurations or through the emulation of more complex schemes via post-selection, the aforementioned witnesses provided experimental evidence for the emergence of an optical dynamics going beyond the passive linear-optical paradigm.

In conclusion, here we have demonstrated a clear gap between the ABS framework and an equivalent BS paradigm in terms of the set of achievable photonic resources, understood as states in the Fock-basis state emerging from an optical interferometer. Crucially, we provided a set of witnesses for the lack of passive separability of a photonic resource. Given the existing link between this concept and bosonic-based quantum computation, our results may be useful as characterization tools in a range of existing photon-based implementations of optical computational tasks. Finally, although large-scale implementations of the ABS paradigm are still out of reach with present photonic technologies, this work highlights how ABS can provide a powerful testbed for exploring nonlinear quantum dynamics going beyond passive linear optics.

## Methods

### Proof of the main theorem about passive separability

As stated in the main text, it can be shown that passively separable states that are eigenstates of the number operator take a specific form.

**Theorem 1**. *For* *m* ≥ 0, *the number operator over* *m* *modes* $\widehat{N}={\sum }_{i=1}^{m}{\widehat{a}}_{i}^{\dagger }{\widehat{a}}_{i}$. *Let* *n* ≥ 0 *and let* $| \psi \rangle$ *be a pure state over* *m* *modes such that* $\widehat{N}| \psi \rangle =n| \psi \rangle$. *Then*, $| \psi \rangle$ *is passive separable if and only if there exists* ${n}_{1},\ldots ,{n}_{m}\in {\mathbb{N}}$ *with* *n*_1_ + ⋯ + *n*_*m*_ = *n* *and an* *m*-*mode interferometer* *U* *such that* $| \psi \rangle =\widehat{U}{\otimes }_{i=1}^{m}| {n}_{i}\rangle$.

***Proof*.** The state $| \psi \rangle =\widehat{U}{\otimes }_{i=1}^{m}| {n}_{i}\rangle$ is intrinsically passively separable by definition. We prove that a passively separable state that is an eigenstate of $\widehat{N}$ necessarily has this form, with ∑*n*_*i*_ = *n*. The state $| \psi \rangle$ satisfies $\hat{\varPi }_{n}^{(m)}| \psi \rangle =| \psi \rangle$, where

6 $${\hat{\varPi }}_{n}^{(m)}:=\mathop{\sum}\limits_{{j}_{1}+\cdots +{j}_{m}=n}| {j}_{1}\rangle \langle {j}_{1}| \otimes \cdots \otimes | {j}_{m}\rangle \langle {j}_{m}|$$

is the projector onto the eigenspace of $\widehat{N}$ for the eigenvalue *n*.

By the definition of passive separability, there exists an *m*-mode interferometer $\widehat{U}$ and single-mode states $| {\psi }_{1}\rangle ,\ldots ,| {\psi }_{m}\rangle$ such that $| \psi \rangle =\widehat{U}{\otimes }_{i=1}^{m}| {\psi }_{i}\rangle$. As ${\widehat{U}}^{\dagger }$ does not change the total number of photons, it commutes with the projector $\hat{\varPi }_{n}^{(m)}$ and thus

7 $$\begin{array}{l}{\hat{\varPi }}_{n}^{(m)}{\otimes}_{i=1}^{m}| {\psi}_{i}\rangle ={\hat{\varPi }}_{n}^{(m)}{\widehat{U}}^{\dagger}| \psi \rangle ={\widehat{U}}^{\dagger}{\hat{\varPi }}_{n}^{(m)}| \psi \rangle \\ ={\widehat{U}}^{\dagger}| \psi \rangle ={\otimes}_{i=1}^{m}| {\psi}_{i}\rangle .\end{array}$$

Writing $| {\psi }_{i}\rangle :={\sum }_{j\ge 0}{\psi }_{i,j}| j\rangle$ in the Fock basis, this directly implies that ${\psi }_{1,{j}_{1}}\cdots {\psi }_{m,{j}_{m}}=0$ whenever *j*_1_ + ⋯ + *j*_*m*_ ≠ *n*. Let *n*_1_, …, *n*_*m*_ such that ${\psi }_{i,{n}_{i}}\ne 0$ for all *i* ∈ {1, …, *m*}. Then *n*_1_ + ⋯ + *n*_*m*_ = *n*, and for all *i* ∈ {1, …, *m*} and all *j* ≠ *n*_*i*_, we have *n*_1_ + ⋯ + *n*_*i*−1_ + *j* + *n*_*i*+1_ + ⋯ + *n*_*m*_ ≠ *n*, so

8 $${\psi }_{1,{n}_{1}}\cdots {\psi }_{i-1,{n}_{i-1}}{\psi }_{i,j}{\psi }_{i+1,{n}_{i+1}}\cdots {\psi }_{m,{n}_{m}}=0,$$

which implies *ψ*_*i*,*j*_ = 0. This shows that $| {\psi }_{i}\rangle =| {n}_{i}\rangle$ for all *i* ∈ {1, …, *m*} and concludes the proof.

### Lie algebraic observables

Following ref. ^34^, we consider Lie algebraic invariants arising from the expectation values of an orthonormal basis of the linear-optical Hamiltonians acting on an *m*-mode system. A natural choice for such a basis is given by the set of Hermitian operators:

9 $$\left\{\begin{array}{ll}{\widehat{O}}_{j}^{z}={\widehat{n}}_{j}={\widehat{a}}_{j}^{\dagger }{\widehat{a}}_{j}, & j=1,\ldots ,m,\\ {\widehat{O}}_{jk}^{x}=\frac{1}{\sqrt{2}}\left({\widehat{a}}_{j}^{\dagger }{\widehat{a}}_{k}+{\widehat{a}}_{k}^{\dagger }{\widehat{a}}_{j}\right), & 1\le j < k\le m,\\ {\widehat{O}}_{jk}^{y}=\frac{i}{\sqrt{2}}\left({\widehat{a}}_{j}^{\dagger }{\widehat{a}}_{k}-{\widehat{a}}_{k}^{\dagger }{\widehat{a}}_{j}\right), & 1\le j < k\le m.\end{array}\right.$$

The expectation values of these observables can be experimentally accessed using multi-mode interferometry. The ${\widehat{O}}_{j}^{z}$ operators correspond to direct population measurements (detecting the number of photons) at the output of the interferometer without further transformations. The ${\widehat{O}}_{jk}^{x,y}$ are reconstructed from the difference in the mean number of photons $\langle {\widehat{n}}_{j}\rangle -\langle {n}_{k}\rangle$ between modes *j* and *k* after the interference between modes *j* and *k* on a balanced beam splitter, with a relative phase of 0 for ${\widehat{O}}_{jk}^{x}$ or π/2 for ${\widehat{O}}_{jk}^{y}$ (ref. ^49^). Diagrams for these measurements are illustrated in the lower right parts of Fig. 5a–c.

We note that the experimental reconstruction of the observables ${\widehat{O}}_{jk}^{x,y}$ relies on the correct implementation of beam splitters and phase shifters. Deviations from these ideal settings result in systematic shifts in the estimated expectation values, leading to a finite precision in the evaluated invariants. The impact of such experimental imperfections on Lie algebraic invariants in linear-optical settings has been analysed in detail in ref. ^40^.

### Experimental apparatus

The experimental apparatus was a state-of-the-art photonic architecture tailored for multi-photon experiments. It comprises several interconnected stages with a high degree of versatility. We employed single-photon sources based on In/GaAs micropillar-embedded quantum-dot emitters^1,50–52^, which were kept at cryogenic temperatures and operated in two different excitation regimes. We refer to the two different sources as QD1 and QD2. For QD1, we employed the so-called longitudinal acoustic phonon-assisted scheme^50^. In this scheme, the excitation laser was slightly blue-detuned with respect to the signal photons, thus making it possible to separate laser residues from the photon signal via a set of narrowband spectral filters^50,53^. Conversely, QD2 was operated in a typical resonant excitation scheme, where the separation between the laser pump and the photon signal was achieved via cross-polarization^51–53^.

For both three- and four-photon experiments, the stream of single photons emitted by the source was fed to an active time-to-spatial demultiplexing system^41,42,54,55^. There, an acousto-optical modulator actively distributed the single-photon stream into up to four output spatial modes with a commutation time of *T*_c_ ≈ 180 ns between each channel. With the addition of properly tuned delay lines, we obtained sequences of up to four time-synchronized photons at the output of the demultiplexing set-up. The average pairwise indistinguishability amongst photons at the output of the demultiplexing set-up was around 83% (90%) for QD1 (QD2), mainly because the photons that constitute the multi-photon state were emitted at temporal distances of hundreds of nanoseconds between each other^42,54^.

The resource states generated were then fed into PICs fabricated with femtosecond laser writing techniques^56^. In the experiments reported in this work, two different PICs were employed, implementing reconfigurable linear-optical networks over either 8 or 12 spatial modes, respectively.

The 8-mode PIC consisted of a rectangular mesh of 28 tunable beam splitters and phase shifters arranged according to the scheme proposed in ref. ^57^, which enabled the implementation of an arbitrary unitary evolution *U* over eight modes^58^. The 12-mode PIC was based on the same architecture and had 66 tunable beam splitters and phase shifters, thus enabling the implementation of an arbitrary unitary evolution *U* over 12 modes.

Reconfigurability in both devices is obtained via thermal phase shifters^59^, whose response was calibrated using coherent laser light at approximately the same wavelength as the single-photon signal^58^. In practice, we achieved an average moduli-fidelity ${{\mathcal{F}}}_{\mathrm{ampl}}=\frac{1}{m}{\sum }_{ij}| {({U}_{t})}_{ij}| | {({U}_{e})}_{ij}|$ between the experimentally implemented on-device transformation *U*_e_ and the expected one *U*_t_ in excess of 0.95, thus confirming our accurate control of the interferometric settings.

## Online content

Any methods, additional references, Nature Portfolio reporting summaries, source data, extended data, supplementary information, acknowledgements, peer review information; details of author contributions and competing interests; and statements of data and code availability are available at 10.1038/s41566-026-01959-3.

## Supplementary information

Supplementary Information Supplementary Sections 1–8 and Figs. 1–8.

## Data Availability

The data that support the finding of this study are reported in the main text and in Supplementary Information. Source datasets are available from the corresponding author upon request.

## References

[1] Heindel, T., Kim, J.-H., Gregersen, N., Rastelli, A. & Reitzenstein, S. Quantum dots for photonic quantum information technology. *Adv. Opt. Photonics* **15**, 613 (2023).

[2] Giordani, T., Hoch, F., Carvacho, G., Spagnolo, N. & Sciarrino, F. Integrated photonics in quantum technologies. *Riv. Nuovo Cimento* **46**, 71–103 (2023).

[3] Wang, J., Sciarrino, F., Laing, A. & Thompson, M. G. Integrated photonic quantum technologies. *Nat. Photon.* **14**, 273–284 (2019).

[4] Aaronson, S. & Arkhipov, A. The computational complexity of linear optics. In *Proc. 43rd Annual ACM Symposium on Theory of Computing* (eds Fortnow, L. & Vadhan, S. P.) 333–342 (Association for Computing Machinery, 2011).

[5] Brod, D. J. et al. Photonic implementation of boson sampling: a review. *Adv. Photonics* **1**, 034001 (2019).

[6] Lund, A. et al. Boson sampling from a Gaussian state. *Phys. Rev. Lett.* **113**, 100502 (2014). 10.1103/PhysRevLett.113.100502 25238340

[7] Hamilton, C. S. et al. Gaussian boson sampling. *Phys. Rev. Lett.* **119**, 170501 (2017). 29219463 10.1103/PhysRevLett.119.170501

[8] Kruse, R. et al. Detailed study of Gaussian boson sampling. *Phys. Rev. A* **100**, 032326 (2019).

[9] Chabaud, U., Markham, D. & Sohbi, A. Quantum machine learning with adaptive linear optics. *Quantum* **5**, 496 (2021).

[10] Spagnolo, N., Brod, D. J., Galvão, E. F. & Sciarrino, F. Non-linear boson sampling. *npj Quantum Inf.* **9**, 3 (2023).

[11] Wang, J. et al. Experimental quantum Hamiltonian learning. *Nat. Phys.* **13**, 551–555 (2017).

[12] Zhong, H.-S. et al. 12-photon entanglement and scalable scattershot boson sampling with optimal entangled-photon pairs from parametric down-conversion. *Phys. Rev. Lett.* **121**, 250505 (2018). 10.1103/PhysRevLett.121.250505 30608840

[13] Wang, H. et al. Boson sampling with 20 input photons and a 60-mode interferometer in a 10^14^-dimensional Hilbert space. *Phys. Rev. Lett.* **123**, 250503 (2019). 10.1103/PhysRevLett.123.250503 31922765

[14] Zhong, H.-S. et al. Quantum computational advantage using photons. *Science* **370**, 1460–1463 (2020). 33273064 10.1126/science.abe8770

[15] Madsen, L. S. et al. Quantum computational advantage with a programmable photonic processor. *Nature* **606**, 75–81 (2022). 35650354 10.1038/s41586-022-04725-x PMC9159949

[16] Deng, Y.-H. et al. Gaussian boson sampling with pseudo-photon-number-resolving detectors and quantum computational advantage. *Phys. Rev. Lett.* **131**, 150601 (2023). 10.1103/PhysRevLett.131.150601 37897783

[17] Hoch, F. et al. Quantum machine learning with adaptive boson sampling via post-selection. *Nat. Commun.* **16**, 902 (2025). 39837818 10.1038/s41467-025-55877-z PMC11751292

[18] Kok, P. et al. Linear optical quantum computing with photonic qubits. *Rev. Mod. Phys.* **79**, 135–174 (2007).

[19] Peyronel, T. et al. Quantum nonlinear optics with single photons enabled by strongly interacting atoms. *Nature* **488**, 57–60 (2012). 22832584 10.1038/nature11361

[20] Englund, D. et al. Ultrafast photon–photon interaction in a strongly coupled quantum dot-cavity system. *Phys. Rev. Lett.* **108**, 093604 (2012). 22463636 10.1103/PhysRevLett.108.093604

[21] Le Jeannic, H. et al. Dynamical photon–photon interaction mediated by a quantum emitter. *Nat. Phys.* **18**, 1191–1195 (2022).

[22] Nielsen, K. H. et al. Programmable nonlinear quantum photonic circuits. *Nat. Commun.* **16**, 11397 (2024). 10.1038/s41467-025-66205-w PMC12738740 41381482

[23] Hansen, L. M. et al. Non-classical excitation of a solid-state quantum emitter. Preprint at https://arxiv.org/abs/2407.20936 (2024).

[24] Knill, E., Laflamme, R. & Milburn, G. J. A scheme for efficient quantum computation with linear optics. *Nature* **409**, 46–52 (2001). 11343107 10.1038/35051009

[25] Scheel, S., Nemoto, K., Munro, W. J. & Knight, P. L. Measurement-induced nonlinearity in linear optics. *Phys. Rev. A* **68**, 032310 (2003).

[26] Raussendorf, R., Browne, D. E. & Briegel, H. J. Measurement-based quantum computation on cluster states. *Phys. Rev. A* **68**, 022312 (2003).

[27] Bartolucci, S. et al. Fusion-based quantum computation. *Nat. Commun.* **14**, 912 (2023). 36805650 10.1038/s41467-023-36493-1 PMC9938229

[28] Crespi, A. et al. Integrated multimode interferometers with arbitrary designs for photonic boson sampling. *Nat. Photonics* **7**, 545–549 (2013).

[29] Bentivegna, M. et al. Experimental scattershot boson sampling. *Sci. Adv.* **1**, e1400255 (2015). 26601164 10.1126/sciadv.1400255 PMC4640628

[30] Zhong, H.-S. et al. Phase-programmable Gaussian boson sampling using stimulated squeezed light. *Phys. Rev. Lett.* **127**, 180502 (2021). 34767431 10.1103/PhysRevLett.127.180502

[31] Migdał, P., Rodríguez-Laguna, J., Oszmaniec, M. & Lewenstein, M. Multiphoton states related via linear optics. *Phys. Rev. A* **89**, 062329 (2014).

[32] Moyano-Fernández, J. J. & Garcia-Escartin, J. C. Linear optics only allows every possible quantum operation for one photon or one port. *Opt. Commun.* **382**, 237–240 (2017).

[33] Garcia-Escartin, J. C., Gimeno, V. & Moyano-Fernández, J. J. Method to determine which quantum operations can be realized with linear optics with a constructive implementation recipe. *Phys. Rev. A* **100**, 022301 (2019).

[34] Parellada, P. V., Garcia, I. V. G., Moyano-Fernández, J. J. & Garcia-Escartin, J. C. Lie algebraic invariants in quantum linear optics. *Quantum* **10**, 2132 (2026).

[35] Mamon, E. Z. Orbit dimensions in linear and Gaussian quantum optics. Preprint at https://arxiv.org/abs/2506.07995 (2025).

[36] Draux, S., Perdrix, S., Jeandel, E. & Mansfield, S. Invariants in linear optics. Preprint at https://arxiv.org/abs/2509.02211 (2025).

[37] Walschaers, M., Fabre, C., Parigi, V. & Treps, N. Entanglement and Wigner function negativity of multimode non-Gaussian states. *Phys. Rev. Lett.* **119**, 183601 (2017). 29219579 10.1103/PhysRevLett.119.183601

[38] Walschaers, M., Fabre, C., Parigi, V. & Treps, N. Statistical signatures of multimode single-photon-added and -subtracted states of light. *Phys. Rev. A* **96**, 053835 (2017).

[39] Albarelli, F., Genoni, M. G., Paris, M. G. A. & Ferraro, A. Resource theory of quantum non-Gaussianity and Wigner negativity. *Phys. Rev. A* **98**, 052350 (2018).

[40] Rodari, G. et al. Observation of Lie algebraic invariants in quantum linear optics. *Phys. Rev. Res.* **7**, 043325 (2025).

[41] Rodari, G. et al. Experimental observation of counter-intuitive features of photonic bunching. *Light Sci. Appl.* **15**, 292 (2026). 42373619 10.1038/s41377-026-02250-4 PMC13315700

[42] Rodari, G. et al. Semi-device independent characterization of multiphoton indistinguishability. *PRX Quantum* **6**, 020340 (2024).

[43] Rodari, G. et al. Polarization-encoded photonic quantum-to-quantum Bernoulli factory based on a quantum dot source. *Sci. Adv.* **10**, eado62 (2024). 10.1126/sciadv.ado6244 PMC11777904 39058770

[44] Schuld, M. & Killoran, N. Quantum machine learning in feature Hilbert spaces. *Phys. Rev. Lett.* **122**, 040504 (2019). 30768345 10.1103/PhysRevLett.122.040504

[45] Chabaud, U. & Mehraban, S. Holomorphic representation of quantum computations. *Quantum* **6**, 831 (2022).

[46] Chabaud, U. & Walschaers, M. Resources for bosonic quantum computational advantage. *Phys. Rev. Lett.* **130**, 090602 (2023). 36930938 10.1103/PhysRevLett.130.090602

[47] Banchi, L., Kolthammer, W. S. & Kim, M. Multiphoton tomography with linear optics and photon counting. *Phys. Rev. Lett.* **121**, 250402 (2018). 30608836 10.1103/PhysRevLett.121.250402

[48] Jozsa, R. Fidelity for mixed quantum states. *J. Mod. Opt.* **41**, 2315–2323 (1994).

[49] Campos, R. A., Saleh, B. E. A. & Teich, M. C. Quantum-mechanical lossless beam splitter: Su(2) symmetry and photon statistics. *Phys. Rev. A* **40**, 1371 (1989). 10.1103/physreva.40.1371 9902272

[50] Thomas, S. E. et al. Bright polarized single-photon source based on a linear dipole. *Phys. Rev. Lett.* **126**, 233601 (2021). 34170172 10.1103/PhysRevLett.126.233601

[51] Somaschi, N. et al. Near-optimal single-photon sources in the solid state. *Nat. Photonics* **10**, 340–345 (2016).

[52] Senellart, P., Solomon, G. & White, A. High-performance semiconductor quantum-dot single-photon sources. *Nat. Nanotechnol.* **12**, 1026–1039 (2017). 29109549 10.1038/nnano.2017.218

[53] Ollivier, H. et al. Reproducibility of high-performance quantum dot single-photon sources. *ACS Photonics* **7**, 1050–1059 (2020).

[54] Pont, M. et al. Quantifying *n*-photon indistinguishability with a cyclic integrated interferometer. *Phys. Rev. X* **12**, 031033 (2022).

[55] Pont, M. et al. High-fidelity four-photon GHz states on chip. *npj Quantum Inf.* **10**, 50 (2024).

[56] Corrielli, G., Crespi, A. & Osellame, R. Femtosecond laser micromachining for integrated quantum photonics. *Nanophotonics* **10**, 3789–3812 (2021).

[57] Clements, W. R., Humphreys, P. C., Metcalf, B. J., Kolthammer, W. S. & Walmsley, I. A. Optimal design for universal multiport interferometers. *Optica* **3**, 1460 (2016).

[58] Pentangelo, C. et al. High-fidelity and polarization-insensitive universal photonic processors fabricated by femtosecond laser writing. *Nanophotonics* **13**, 2259–2270 (2024). 39634510 10.1515/nanoph-2023-0636 PMC11501604

[59] Albiero, R. et al. Toward higher integration density in femtosecond-laser-written programmable photonic circuits. *Micromachines* **13**, 1145 (2022). 35888962 10.3390/mi13071145 PMC9320504

