# Supplementary material for: Beyond quantum linear optics with adaptive boson sampling
Source: Nat Photonics. 2026 Jul 20;20(8):941–9. doi: 10.1038/s41566-026-01959-3 (PMC13427631; doi:10.1038/s41566-026-01959-3)
Supplement: Supplementary file 1 — Supplementary Sections 1–8 and Figs. 1–8. [file 41566_2026_1959_MOESM1_ESM.pdf]

nature photonics

Supplementary information https://doi.org/10.1038/s41566-026-01959-3

# Beyond quantum linear optics with adaptive boson sampling

In the format provided by the authors and unedited

## Contents

1. 2-photon, 2-mode output state tomography 2

2. Details on the real-time adaptive experiment 5

3. Details on the numerical model of the experiment 6

4. Effect of adaptive unitaries on the fidelity kernel 7

5. Minimum trace distance with linear optics states 7

6. Details on post-selected ABS experiments for the measurement of Lie invariant quantities 9
   A. Single-stage ABS Experiments 9
   B. Two-stage ABS Experiments 11

7. Witnessing non-linear dynamics from the spectrum of the Projection of the Density Matrix onto the Subalgebra of Linear Optical Hamiltonians 13

8. Measuring Lie invariant quantities for mixed ABS outputs 14

## Supplementary Note 1. 2-photon, 2-mode output state tomography

In this Supplementary Note we explain the tomography procedure followed to reconstruct the output state of two photons in two modes. This state lives in a three-dimensional Hilbert space spanned by the basis $\{|2,0\rangle, |1,1\rangle, |0,2\rangle\}$, and is therefore treated as a qutrit. Its tomography is performed by measuring the expectation value of the generalized Pauli operators $\{\hat{\sigma}_i\}$[1, 2]:

$$\hat{\sigma}_1 \doteq \begin{bmatrix} 0 & 1 & 0 \\ 1 & 0 & 0 \\ 0 & 0 & 0 \end{bmatrix} \quad \hat{\sigma}_2 \doteq \begin{bmatrix} 0 & -i & 0 \\ i & 0 & 0 \\ 0 & 0 & 0 \end{bmatrix} \quad \hat{\sigma}_3 \doteq \begin{bmatrix} 1 & 0 & 0 \\ 0 & -1 & 0 \\ 0 & 0 & 0 \end{bmatrix} \quad \hat{\sigma}_4 \doteq \begin{bmatrix} 0 & 0 & 1 \\ 0 & 0 & 0 \\ 1 & 0 & 0 \end{bmatrix}$$
$$\hat{\sigma}_5 \doteq \begin{bmatrix} 0 & 0 & -i \\ 0 & 0 & 0 \\ i & 0 & 0 \end{bmatrix} \quad \hat{\sigma}_6 \doteq \begin{bmatrix} 0 & 0 & 0 \\ 0 & 0 & 1 \\ 0 & 1 & 0 \end{bmatrix} \quad \hat{\sigma}_7 \doteq \begin{bmatrix} 0 & 0 & 0 \\ 0 & 0 & -i \\ 0 & i & 0 \end{bmatrix} \quad \hat{\sigma}_8 \doteq \frac{1}{\sqrt{3}} \begin{bmatrix} 1 & 0 & 0 \\ 0 & 1 & 0 \\ 0 & 0 & 2 \end{bmatrix}, \tag{1}$$

which, in the case of a qutrit, correspond to the well-known Gell-Mann matrices.

The density matrix is reconstructed from the expectation values $\langle \hat{\sigma}_i \rangle$ as:

$$\hat{\rho} = \frac{1}{3}\hat{\mathbb{I}} + \frac{1}{2}\sum_{i=1}^{8} \langle \hat{\sigma}_i \rangle \hat{\sigma}_i. \tag{2}$$

To extract the expectation values $\langle \hat{\sigma}_i \rangle$, we apply a set of 9 tomography unitaries $\{T^m\}$ prior to measurement. Each unitary corresponds to specific values of internal and external phases $(\theta_m, \phi_m)$ and transforms the state $\hat{\rho}$ before projection. The outcomes are measured in the $\{|2,0\rangle, |0,2\rangle, |1,1\rangle\}$ basis, labeled by $l = 0, 1, 2$. The unitaries are designed so that each Pauli operator can be expressed as a linear combination of the associated projectors:

$$\hat{\sigma}_i = \sum_{m,l} c^i_{ml} \hat{\Pi}_{ml}, \tag{3}$$

yielding the expectation values as:

$$\langle \hat{\sigma}_i \rangle = \mathrm{Tr}(\hat{\sigma}_i \hat{\rho}) = \sum_{m,l} c^i_{ml} p_{ml}, \tag{4}$$

where $p_{ml} = \mathrm{Tr}(\hat{\Pi}_{ml}\hat{\rho})$ is the measured probability.

In the following the 9 chosen tomography unitaries are presented, showing for each one the values of the internal and external phases $(\theta_m, \phi_m)$ and the matrix describing the corresponding unitary transformation $\hat{T}^m$ in the $\{|2,0\rangle, |0,2\rangle, |1,1\rangle\}$ basis.

$$
(\theta_0,\phi_0)=(0,0)\implies \hat{T}^0=\begin{bmatrix}1&0&0\\0&1&0\\0&0&-1\end{bmatrix}
\qquad
(\theta_1,\phi_1)=\left(\frac{\pi}{2},0\right)\implies \hat{T}^1=\begin{bmatrix}\frac12&\frac12&\frac{1}{\sqrt2}\\ \frac12&\frac12&-\frac{1}{\sqrt2}\\ \frac{1}{\sqrt2}&-\frac{1}{\sqrt2}&0\end{bmatrix}
$$

$$
(\theta_2,\phi_2)=\left(\frac{\pi}{2},\frac{\pi}{2}\right)\implies \hat{T}^2=\begin{bmatrix}-\frac12&\frac12&\frac{i}{\sqrt2}\\ -\frac12&\frac12&-\frac{i}{\sqrt2}\\ -\frac{1}{\sqrt2}&-\frac{1}{\sqrt2}&0\end{bmatrix}
\qquad
(\theta_3,\phi_3)=\left(\frac{\pi}{4},0\right)\implies \hat{T}^3=\begin{bmatrix}\frac{2+\sqrt2}{4}&\frac{2-\sqrt2}{4}&\frac12\\ \frac{2-\sqrt2}{4}&\frac{2+\sqrt2}{4}&-\frac12\\ \frac12&-\frac12&\frac{1}{\sqrt2}\end{bmatrix}
$$

$$
(\theta_4,\phi_4)=\left(\frac{\pi}{4},\pi\right)\implies \hat{T}^4=\begin{bmatrix}\frac{2+\sqrt2}{4}&\frac{2-\sqrt2}{4}&-\frac12\\ \frac{2-\sqrt2}{4}&\frac{2+\sqrt2}{4}&\frac12\\ \frac12&-\frac12&\frac{1}{\sqrt2}\end{bmatrix}
\qquad
(\theta_5,\phi_5)=\left(\frac{\pi}{2},\frac{\pi}{4}\right)\implies \hat{T}^5=\begin{bmatrix}\frac{i}{2}&\frac12&\frac{1+i}{2}\\ \frac{i}{2}&\frac12&\frac{-1-i}{2}\\ \frac{i}{\sqrt2}&-\frac{1}{\sqrt2}&0\end{bmatrix}
$$

$$
(\theta_6,\phi_6)=\left(\frac{\pi}{2},-\frac{\pi}{4}\right)\implies \hat{T}^6=\begin{bmatrix}-\frac{i}{2}&\frac12&\frac{1-i}{2}\\ -\frac{i}{2}&\frac12&\frac{i-1}{2}\\ -\frac{i}{\sqrt2}&-\frac{1}{\sqrt2}&0\end{bmatrix}
\qquad
(\theta_7,\phi_7)=\left(\frac{\pi}{4},\frac{\pi}{2}\right)\implies \hat{T}^7=\begin{bmatrix}-\frac{2+\sqrt2}{4}&\frac{2-\sqrt2}{4}&\frac{i}{2}\\ -\frac{2-\sqrt2}{4}&\frac{2+\sqrt2}{4}&-\frac{i}{2}\\ -\frac12&-\frac12&-\frac{i}{\sqrt2}\end{bmatrix}
$$

$$
(\theta_8,\phi_8)=\left(\frac{\pi}{4},-\frac{\pi}{2}\right)\implies \hat{T}^8=\begin{bmatrix}-\frac{2+\sqrt2}{4}&\frac{2-\sqrt2}{4}&-\frac{i}{2}\\ -\frac{2-\sqrt2}{4}&\frac{2+\sqrt2}{4}&\frac{i}{2}\\ -\frac12&-\frac12&\frac{i}{\sqrt2}\end{bmatrix}. \tag{5}
$$

The matrices are obtained using the following convention for a unitary transformation in the $\{|1,0\rangle, |0,1\rangle\}$ basis:

$$
T(\theta,\phi)=\begin{bmatrix}\cos\frac{\theta}{2}e^{i\phi}&\sin\frac{\theta}{2}\\ \sin\frac{\theta}{2}e^{i\phi}&-\cos\frac{\theta}{2}\end{bmatrix}, \tag{6}
$$

which in the $\{|2,0\rangle, |0,2\rangle, |1,1\rangle\}$ basis corresponds to the transformation:

$$
\hat{T}(\theta,\phi)=\begin{bmatrix}\cos^2\frac{\theta}{2}e^{2i\phi}&\sin^2\frac{\theta}{2}&\sqrt2\cos\frac{\theta}{2}\sin\frac{\theta}{2}e^{i\phi}\\ \sin^2\frac{\theta}{2}e^{2i\phi}&\cos^2\frac{\theta}{2}&-\sqrt2\cos\frac{\theta}{2}\sin\frac{\theta}{2}e^{i\phi}\\ \sqrt2\cos\frac{\theta}{2}\sin\frac{\theta}{2}e^{2i\phi}&-\sqrt2\cos\frac{\theta}{2}\sin\frac{\theta}{2}&(-\cos^2\frac{\theta}{2}+\sin^2\frac{\theta}{2})e^{i\phi}\end{bmatrix}. \tag{7}
$$

The output probabilities for the action of each unitary transformation on a given state $\hat{\rho}$ are:

$$
\begin{cases}p_{m0}=\langle\psi_{m0}|\,\hat{\rho}\,|\psi_{m0}\rangle\\ p_{m1}=\langle\psi_{m1}|\,\hat{\rho}\,|\psi_{m1}\rangle\\ p_{m2}=\langle\psi_{m2}|\,\hat{\rho}\,|\psi_{m2}\rangle\end{cases}
\qquad
\begin{cases}\langle\psi_{m0}|=\hat{T}^m_{00}\,\langle 2,0|+\hat{T}^m_{01}\,\langle 0,2|+\hat{T}^m_{02}\,\langle 1,1|\\ \langle\psi_{m1}|=\hat{T}^m_{10}\,\langle 2,0|+\hat{T}^m_{11}\,\langle 0,2|+\hat{T}^m_{12}\,\langle 1,1|\\ \langle\psi_{m2}|=\hat{T}^m_{20}\,\langle 2,0|+\hat{T}^m_{21}\,\langle 0,2|+\hat{T}^m_{22}\,\langle 1,1|\end{cases} \tag{8}
$$

So knowing the elements of each unitary matrix $\hat{T}^m$ one can obtain the states $\{|\psi_{ml}\rangle\}$ and thus the projectors $\{\hat{\Pi}_{ml}\}$:

$$
\hat{\Pi}_{ml}=|\psi_{ml}\rangle\langle\psi_{ml}|\,. \tag{9}
$$

It is possible to show that, with our choice of the phase settings, the $\{\hat{\sigma}_i\}$ operators can be then obtained from the

projectors $\hat{\Pi}_{ml}$ with the following linear combinations:

$$\begin{cases} \hat{\sigma}_1 = \hat{\Pi}_{22} - \hat{\Pi}_{12} \\ \hat{\sigma}_2 = \hat{\Pi}_{62} - \hat{\Pi}_{52} \\ \hat{\sigma}_3 = \hat{\Pi}_{00} - \hat{\Pi}_{01} \\ \hat{\sigma}_4 = \frac{1}{\sqrt{2}}(\hat{\Pi}_{10} - \hat{\Pi}_{11} - \hat{\Pi}_{32} + \hat{\Pi}_{42}) \\ \hat{\sigma}_5 = \frac{1}{\sqrt{2}}(\hat{\Pi}_{82} - \hat{\Pi}_{72} - \hat{\Pi}_{21} + \hat{\Pi}_{20}) \\ \hat{\sigma}_6 = \frac{1}{\sqrt{2}}(\hat{\Pi}_{10} - \hat{\Pi}_{11} + \hat{\Pi}_{32} - \hat{\Pi}_{42}) \\ \hat{\sigma}_7 = \frac{1}{\sqrt{2}}(\hat{\Pi}_{82} - \hat{\Pi}_{72} + \hat{\Pi}_{21} - \hat{\Pi}_{20}) \\ \hat{\sigma}_8 = \frac{1}{\sqrt{3}}(\hat{\Pi}_{00} + \hat{\Pi}_{01} - 2\hat{\Pi}_{02}) \end{cases} \tag{10}$$

Therefore, the set of projectors $\{\hat{\Pi}_{ml}\}$ is sufficient to give full information about the output quantum state.

In the real-time adaptive experiment, the two output modes are encoded in the polarization degree of freedom to perform tomography of the output state. Specifically, the spatial modes exiting the displaced Sagnac interferometer are mapped onto the horizontal and vertical polarization states, such that the single-photon basis $\{|1,0\rangle, |0,1\rangle\}$ corresponds to $\{|H\rangle, |V\rangle\}$. In this representation, the tomography unitaries $T(\theta, \phi)$ are implemented using polarization optics placed after the adaptive stage.

The action of the polarization elements is described within the Jones formalism [3]. In particular, we employ a quarter-wave plate (QWP) and a half-wave plate (HWP) rotated by an angle $\theta$ with respect to the optical axis. Their Jones matrices read

$$Q(\theta) = e^{-i\pi/4} \begin{pmatrix} \cos^2\theta + i\sin^2\theta & (1-i)\sin\theta\cos\theta \\ (1-i)\sin\theta\cos\theta & \sin^2\theta + i\cos^2\theta \end{pmatrix}, \tag{11}$$

$$H(\theta) = \begin{pmatrix} \cos 2\theta & \sin 2\theta \\ \sin 2\theta & -\cos 2\theta \end{pmatrix}. \tag{12}$$

The overall polarization transformation implemented in the experiment is therefore given by

$$U(\theta_{\mathrm{QWP}}, \theta_{\mathrm{HWP}}) = H(\theta_{\mathrm{HWP}})\, Q(\theta_{\mathrm{QWP}}). \tag{13}$$

For each tomography setting $(\theta_m, \phi_m)$, the angles $(\theta_{\mathrm{QWP}}, \theta_{\mathrm{HWP}})$ are determined numerically such that the transformation $U(\theta_{\mathrm{QWP}}, \theta_{\mathrm{HWP}})$ reproduces the target unitary $T(\theta_m, \phi_m)$ introduced above. The optimization is performed up to independent phase factors on each output mode, i.e.

$$U(\theta_{\mathrm{QWP}}, \theta_{\mathrm{HWP}}) = D\, T(\theta_m, \phi_m), \tag{14}$$

where $D$ is a diagonal matrix containing phase factors. These phases do not affect the tomography procedure, since the experimentally relevant quantities are the projectors $\hat{\Pi}_{ml} = |\psi_{ml}\rangle\langle\psi_{ml}|$, which are invariant under such row-dependent phase shifts. With this procedure, the same set of tomographic transformations $\{\hat{T}^m\}$ introduced above can be implemented in the polarization encoding. The corresponding projective measurements are then obtained by measuring the photons after the wave plate sequence and projecting onto the $\{|HH\rangle, |VV\rangle, |HV\rangle\}$ basis.

Because of the limited number of samples obtained experimentally for each observable, each quantum state is actually reconstructed from the outcomes of the tomography projective measurements with a Maximum-Likelihood approach. This consists in finding, among all possible density matrices describing the state, the one which maximizes the probability of obtaining the experimental output probabilities $\{p_{ml}\}$ [4]. We show in Supplementary Fig. 1-(a) examples of reconstructed density matrices from the ABS experiment with $n = 4$, $n' = 2$, $m' = 2$, where adaptivity is emulated through post-selection, while in Supplementary Fig. 1-(b) we show examples of experimentally reconstructed density matrices obtained via Maximum-Likelihood from the real-time ABS experiment in the $n = 3$, $n' = 2$, $m' = 2$ configuration.

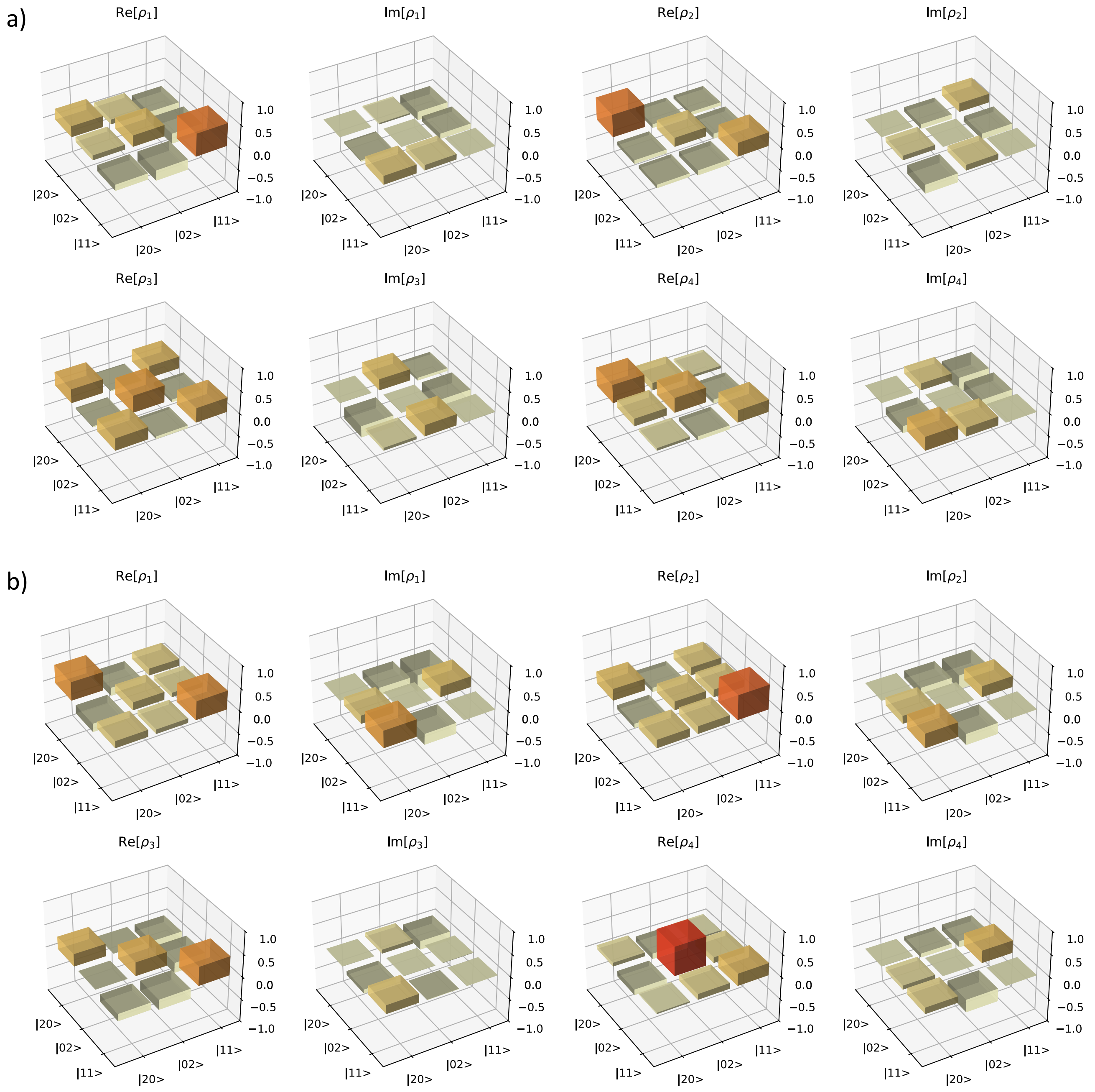


Supplementary Figure 1. **3D plots of the real and imaginary parts of reconstructed density matrices via Maximum-Likelihood from the ABS experiment in the $n' = 2$, $m' = 2$ scenario.** a) Results obtained in the $n = 4$ experiment, where adaptivity is emulated through post-selection. b) Results obtained from the $n = 3$ real-time adaptive experiment.

## Supplementary Note 2. Details on the real-time adaptive experiment

In this Supplementary Note we provide a detailed description of the optical apparatus used to implement the real-time adaptive ABS experiment, as schematically illustrated in Supplementary Fig. 2. The setup is based on an 8-mode programmable integrated photonic circuit that first implements an initial unitary $U_0$ acting on a three-photon input state. Six of the output modes are detected by single-photon avalanche photodiodes (APDs), while the remaining two modes are routed to the adaptive processing stage.

A path-to-polarization conversion enables the polarization encoding of the unmeasured two-photon state. Sets of waveplates are mounted on both branches to optimize the signal at the output of the polarizing beam splitter, and the relative phase between the branches is actively stabilized through an auxiliary photon counter-propagating

through the chip. The counter-propagating photon is detected by superconducting nanowire single-photon detectors (SNSPDs), and the resulting signal provides feedback to a proportional–integral–derivative control loop acting on a piezo-electric element controlling the orientation of a mirror.

The polarization-encoded state is then delayed with respect to the APD channels using a 240 m-long optical fiber, providing approximately 1.2 $\mu$s of temporal separation to allow for electronic feed-forward processing. A polarization controller compensates any polarization rotation introduced by the fiber. After this delay, the two-photon state is converted back to spatial encoding via a displaced Sagnac interferometer. At this stage, a fast electro-optic phase modulator applies a measurement-conditioned phase shift, where the applied voltage depends on the APD-detected outcome $\boldsymbol{p}$, thereby realizing an outcome-conditioned unitary transformation $V(\boldsymbol{p})$.

Finally, the output state is reconstructed using projective measurements implemented with a combination of quarter-wave and half-wave plates (performing tomographic measurement projections), a polarizing beam splitter, and two fiber beam splitters for a pseudo-number-resolving detection scheme. Two-fold photon coincidences are detected by SNSPDs and recorded using a time-to-digital converter. The APDs employed in the adaptive experiments operate at a count rate of approximately 500 kHz, and the electro-optic modulator is driven at the same repetition rate, ensuring real-time implementation of adaptive operations within the fiber delay window.

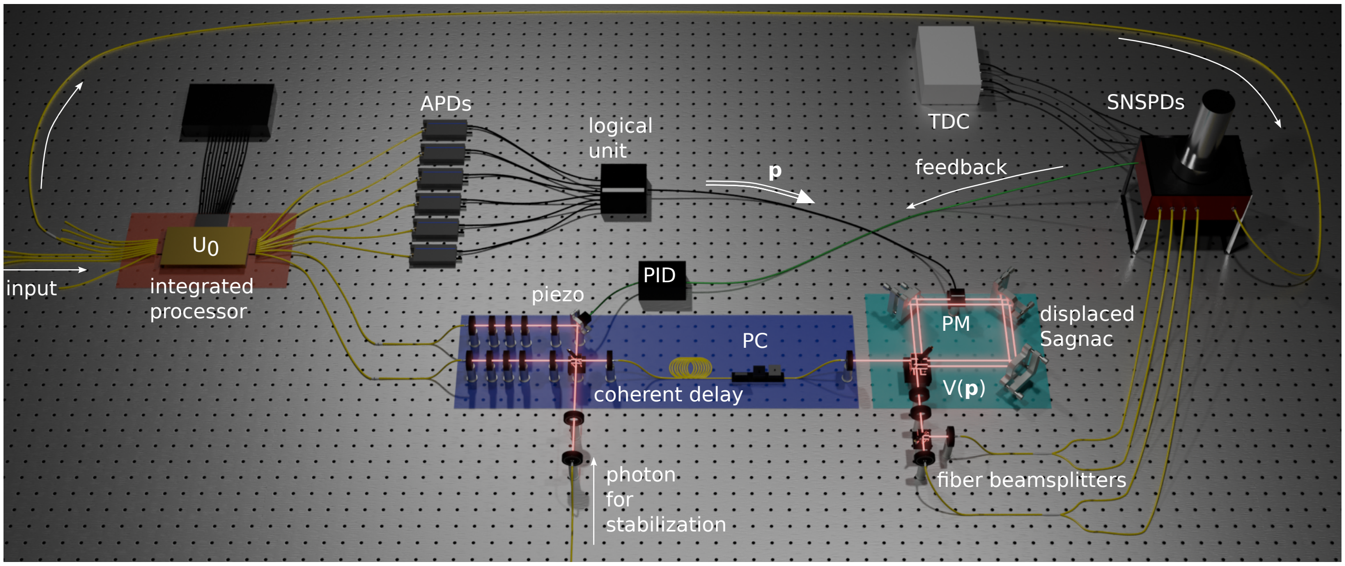


Supplementary Figure 2. **Experimental scheme for real-time adaptive measurements and feed-forward operations.** A three-photon input state is processed by an 8-mode integrated processor, with six modes monitored by avalanche photodiodes (APDs), where the detection of a single photon is performed, and the remaining two modes routed to the adaptive stage. The resulting two-photon state is encoded in the polarization degree of freedom, with active phase stabilization implemented using a counter-propagating photon and a proportional–integral–derivative (PID) control loop acting on a piezo-electric element. Coherent delay is provided by a 240 m-long optical fiber, compensated with a polarization controller (PC). The two-photon state is converted back to spatial encoding via a displaced Sagnac interferometer. Here the adaptive transformation $V(\boldsymbol{p})$ is realized using a logical unit to process measurement outcomes and control a fast phase modulator (PM) acting on one interferometer arm. The final state is measured using wave plates, a polarizing beam splitter, and fiber beam splitters in a pseudo-number-resolving scheme, with two-fold coincidences recorded by superconducting nanowire single-photon detectors (SNSPDs) via a time-to-digital converter (TDC).

## Supplementary Note 3. Details on the numerical model of the experiment

In this Supplementary Note we briefly describe the numerical model used to compute the simulated output probability distributions $\boldsymbol{P}_{\mathrm{sim}}$ introduced in the main text. The model accounts for the dominant non-idealities of the experimental platform, namely pairwise partial photon distinguishability in the multi-photon state prepared by the demultiplexing stage, multi-photon emission from the quantum dot source, and optical losses.

Partial photon distinguishability is incorporated following the formalism introduced in [5], where many-photon interference is described in terms of a Gram matrix $S$, with elements $S_{i,j} = \langle\psi_i|\psi_j\rangle$ encoding the pairwise overlaps between the internal states of the photons. This approach allows us to evaluate output probabilities beyond the perfectly indistinguishable regime.

Multi-photon emission from the quantum dot source, instead, is modeled by assuming that each input mode is described by a mixed state:

$$\rho = p_0 \left|0\right\rangle \left\langle 0\right| + p_1 \left|1\right\rangle \left\langle 1\right| + p_2 \left|1, \tilde{1}\right\rangle \left\langle 1, \tilde{1}\right| , \tag{15}$$

where $|1\rangle$ denotes the desired single-photon component and $|1, \tilde{1}\rangle$ represents a two-photon contribution including an additional noise photon assumed to be fully distinguishable from the principal one. The parameters $p_0$, $p_1$ and $p_2$ are determined from the measured source brightness and $g^{(2)}(0)$ [6].

Optical losses are assumed to be approximately balanced across all modes of the interferometer. Under this assumption, losses commute with passive linear-optical transformations and can be modeled as a uniform transmission acting on each photon.

Importantly, the numerical simulations assume ideal implementations of the unitary transformation describing the input-output transformation.

## Supplementary Note 4. Effect of adaptive unitaries on the fidelity kernel

In the main text, fidelity kernel matrices are used to visualize the relative distribution of Adaptive Boson Sampling (ABS) output states within the output Fock space. For a given experiment, the kernel is defined as

$$K_{ij} = \left( \mathrm{Tr} \sqrt{\sqrt{\hat{\rho}_i}\, \hat{\rho}_j \, \sqrt{\hat{\rho}_i}} \right)^2 , \tag{16}$$

where $\hat{\rho}_i$ and $\hat{\rho}_j$ denote the reconstructed conditional output states corresponding to different adaptive outcomes.

In our implementation of ABS, a fixed number $M$ of measurement outcomes $\boldsymbol{p}_i$ is selected, each associated with an adaptive unitary $V_i$. While the main text presents kernel matrices obtained from a single such adaptive map, since the experiment is performed in post-selection, the same experimental data allow for multiple consistent adaptive evolutions obtained by reassigning the unitaries $V_i$ to the measurement outcomes $\boldsymbol{p}_i$.

Specifically, we can generate additional kernels by performing cyclic permutations of the $(\boldsymbol{p}_i, V_i)$ pairs,

$$(\boldsymbol{p}_1, V_1), (\boldsymbol{p}_2, V_2), \ldots, (\boldsymbol{p}_M, V_M) \;\rightarrow\; (\boldsymbol{p}_1, V_2), (\boldsymbol{p}_2, V_3), \ldots, (\boldsymbol{p}_M, V_1), \tag{17}$$

and iterating this procedure $M$ times. Each permutation defines a distinct adaptive evolution and yields a new set of conditional output states, from which a corresponding fidelity kernel can be reconstructed. This procedure avoids redundancy while systematically exploring the space of possible adaptive maps. The first kernel (no permutation applied) coincides with the kernel shown in the main text.

Supplementary Fig. 3 reports all kernels obtained in this way for the $n = 4$, $n' = 2, m' = 2$ experiment where adaptivity is emulated through post-selection, corresponding to $M = 15$ adaptive outcomes and thus 15 different kernels. Although all kernels are derived from the same experimental data, they exhibit different structures, reflecting distinct distributions of the ABS output states in the output Hilbert space.

## Supplementary Note 5. Minimum trace distance with linear optics states

We now introduce an alternative method to quantify the difference between output states produced by various ABS protocols and those generated via linear optics, assuming the same number of photons and modes in both cases. This method uses the trace distance:

$$\mathrm{TD}(\hat{\rho}, \hat{\sigma}) = \frac{1}{2} \mathrm{Tr} |\hat{\rho} - \hat{\sigma}|, \tag{18}$$

which can be estimated from the output state populations (i.e., the output distributions) only. Our goal is to characterize the distribution of minimum trace distances between ABS states and linear optics states.

To this end, we generate a reference set of 20000 linear optics states by sampling Haar-random unitaries in $SU(m-k)$ with two different fixed input states for each configuration: 10000 with input state $|1, 1, 0, \ldots\rangle$ and 10000 with input $|2, 0, 0, \ldots\rangle$. For each ABS state, we compute the trace distance to all linear optics states and record the minimum value. We repeat this procedure across two different configurations:

(a) $n' = 2$ photons in $m' = 3$ modes ($D = 6$);

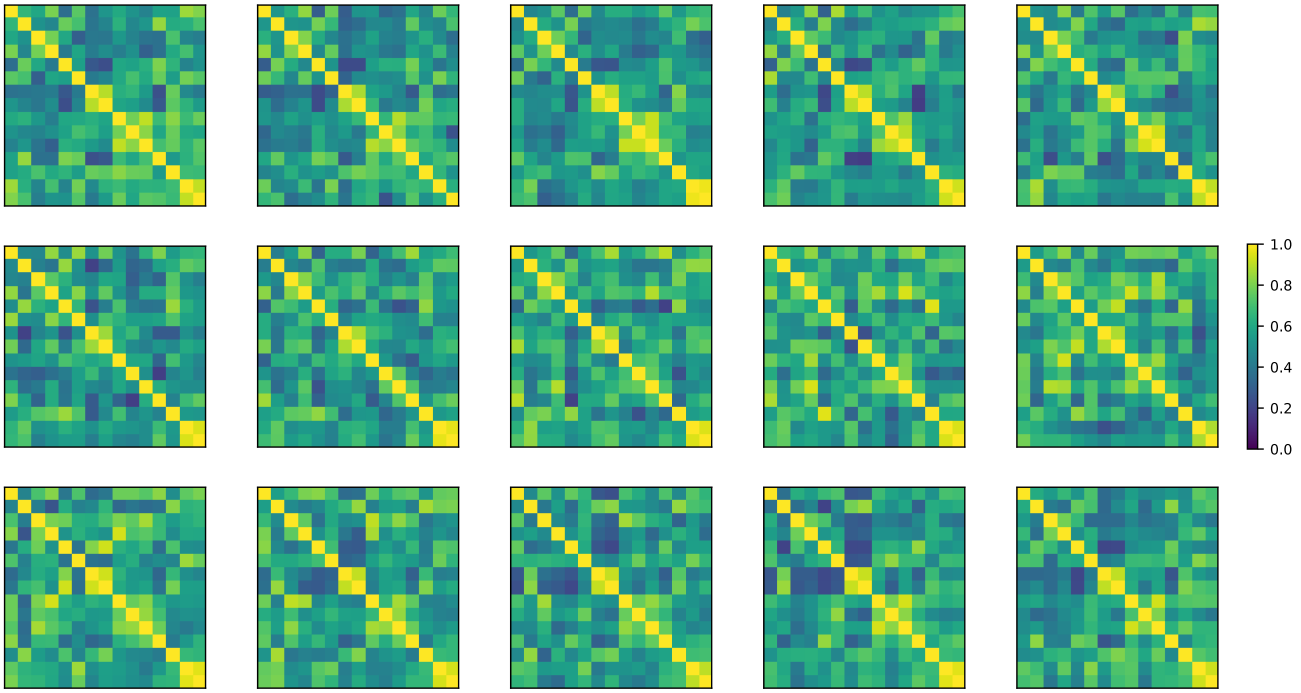


Supplementary Figure 3. **Effect of adaptivity on ABS output fidelity kernels.** Fidelity kernel matrices $K_{ij} = \left(\mathrm{Tr}\sqrt{\sqrt{\hat{\rho}_i}\hat{\rho}_j\sqrt{\hat{\rho}_i}}\right)^2$ reconstructed from experimentally obtained ABS output states in the post-selected $m' = 2, n' = 2$ experiment, for different adaptive maps between $M = 15$ measurement outcomes $\boldsymbol{p}_i$ and adaptive unitaries $V_i$. Each kernel is obtained by cyclically permuting the correspondence between the set of measurement outcomes and the adaptive unitaries. The first kernel corresponds to the adaptive map used in the main text, while the other kernels are generated by successive cyclic permutations. The structure of the fidelity kernels is visibly modified under these permutations, demonstrating that adaptive unitaries play an operational role in shaping the distribution of ABS output states.

(b) $n' = 2$ photons in $m' = 4$ modes ($D = 10$).

In each case, we compute the distribution of minimum trace distances for several classes of states:

- random ABS states,
- experimentally obtained ABS states,
- random pure states in the corresponding Hilbert space (i.e., random qudits of dimension $D$),
- additional random linear optics states (distinct from the reference set).

The results are shown in Supplementary Fig. 4, where panels (a) and (b) correspond to the two configurations above. For each configuration the statistical data for the experimentally obtained ABS states is increased by considering again permutations in the association of the measurement outcome $\boldsymbol{p}_i$ and the adaptive unitary $V_i$, since these data are obtained from experiments performed by emulating adaptivity via post-selection. As expected, the distribution of minimum distances for linear optics states compared to other linear optics states is peaked around zero, indicating that these states can be well approximated within the same class. In contrast, both random and experimental ABS states tend to exhibit larger minimum distances, suggesting that many ABS states cannot be generated by linear optics alone. Interestingly, the distribution for ABS states remains similar to that of fully random states in the corresponding Hilbert space. However, we observe that this distinction becomes less pronounced as the number of modes increases, indicating that the trace distance may offer a weaker witness of non-linear dynamics in higher-dimensional settings.

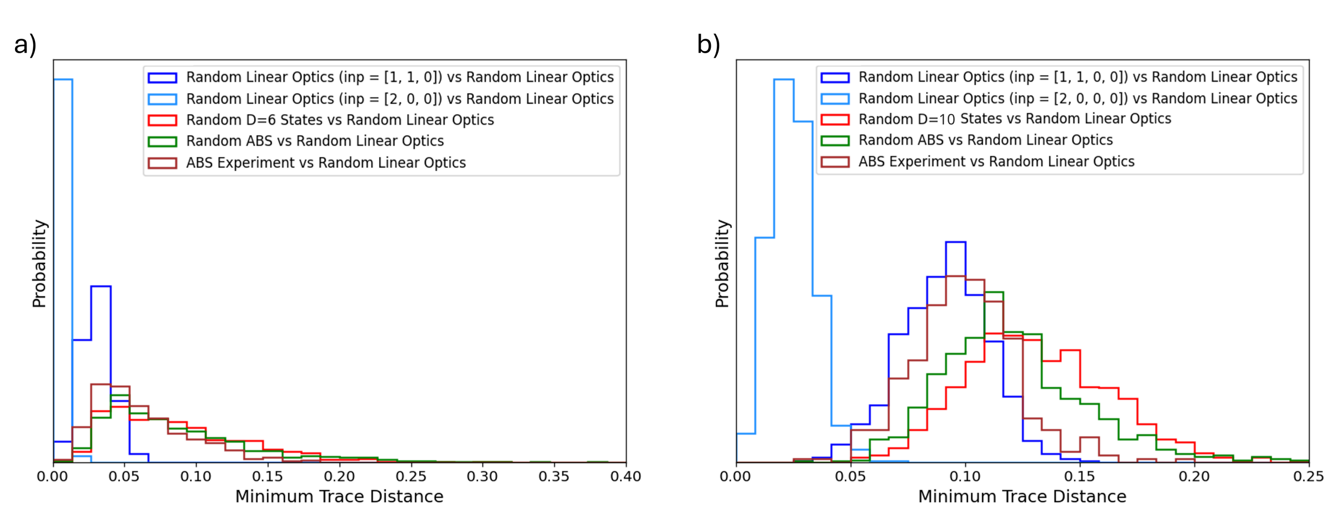


Supplementary Figure 4. **Histograms of the minimum trace distance between linear-optics states and different sets of states.** The histograms include random ABS states, experimental ABS states, random qudit states of the same Hilbert space dimension, and other random linear optics states. Each minimum distance is computed relative to a sample of 20000 Haar-random linear optics states. (a) $n' = 2$, $m' = 3$ ($D = 6$); (b) $n' = 2$, $m' = 4$ ($D = 10$).

## Supplementary Note 6. Details on post-selected ABS experiments for the measurement of Lie invariant quantities

In this Supplementary Note we provide more details regarding the experiments done for measuring Lie invariant quantities, focusing in particular on the evaluation of the quantity $I(\hat{\rho}) = \sum_{i=1}^{m^2} \left(\mathrm{Tr}(\hat{O}_i \hat{\rho})\right)^2$ as defined in the main text.

### A. Single-stage ABS Experiments

We provide here additional details on the experimental implementation of the single-stage Adaptive Boson Sampling (ABS) protocols employed for the measurement of Lie invariant quantities. All the experiments discussed in this section are implemented by emulating adaptivity through post-selection.

Two distinct classes of single-stage ABS experiments are considered. The first class corresponds to configurations with $n' = 2$ and $m' = 2$, obtained from the evolution of $n = 3$ or $n = 4$ input photons through an 8-mode interferometer $U_0$, followed by a two-mode adaptive unitary $V$ conditioned on the intermediate measurement outcomes. For the case $n = 3$, these experiments correspond to an independent set of post-selected ABS implementations not discussed in the main text, while for $n = 4$ the same experimental data as those used to construct the post-selected kernel shown in the main text are considered. In all cases, the Lie invariant $I(\hat{\rho}_{\mathrm{ABS}})$ is evaluated by measuring the expectation values of the corresponding Lie observables $\hat{O}_i$, without relying on full state tomography.

The second class of experiments includes configurations with $(n', m') = (2, 3), (2, 4), (3, 3)$ and $(3, 4)$. In these cases, four photons are injected into an initial 8-mode interferometer $U_0$, and a subset of the photons is detected at the intermediate measurement stage, leaving $n'$ photons propagating in the remaining $m'$ unmeasured modes. These modes subsequently evolve under a single adaptive unitary $V$. After this evolution, additional linear-optical transformations are again applied in order to measure the expectation values of the Lie observables $\hat{O}_i$ required to compute the invariant $I(\hat{\rho}_{\mathrm{ABS}})$. The corresponding interferometric architecture is schematically illustrated in Supplementary Fig. 5-(a) for a representative case ($n' = 3$, $m' = 3$).

The distributions of the experimentally measured values of the invariant $I(\hat{\rho}_{\mathrm{ABS}})$ for all investigated single-stage ABS configurations are shown in Supplementary Fig. 5-(d). Since the experiments are performed in a post-selection regime, the collected data can be reinterpreted by considering different permutations between the set of $M$ adaptive unitaries and the $M$ possible measurement outcomes $\boldsymbol{p}_i$. The reported histograms therefore aggregate the results obtained from $M^2$ distinct effective output states, each time for three different choices of the initial unitary $U_0$.

To assess the reliability of the experimental implementation in this regime, Supplementary Fig. 5-(b) reports the

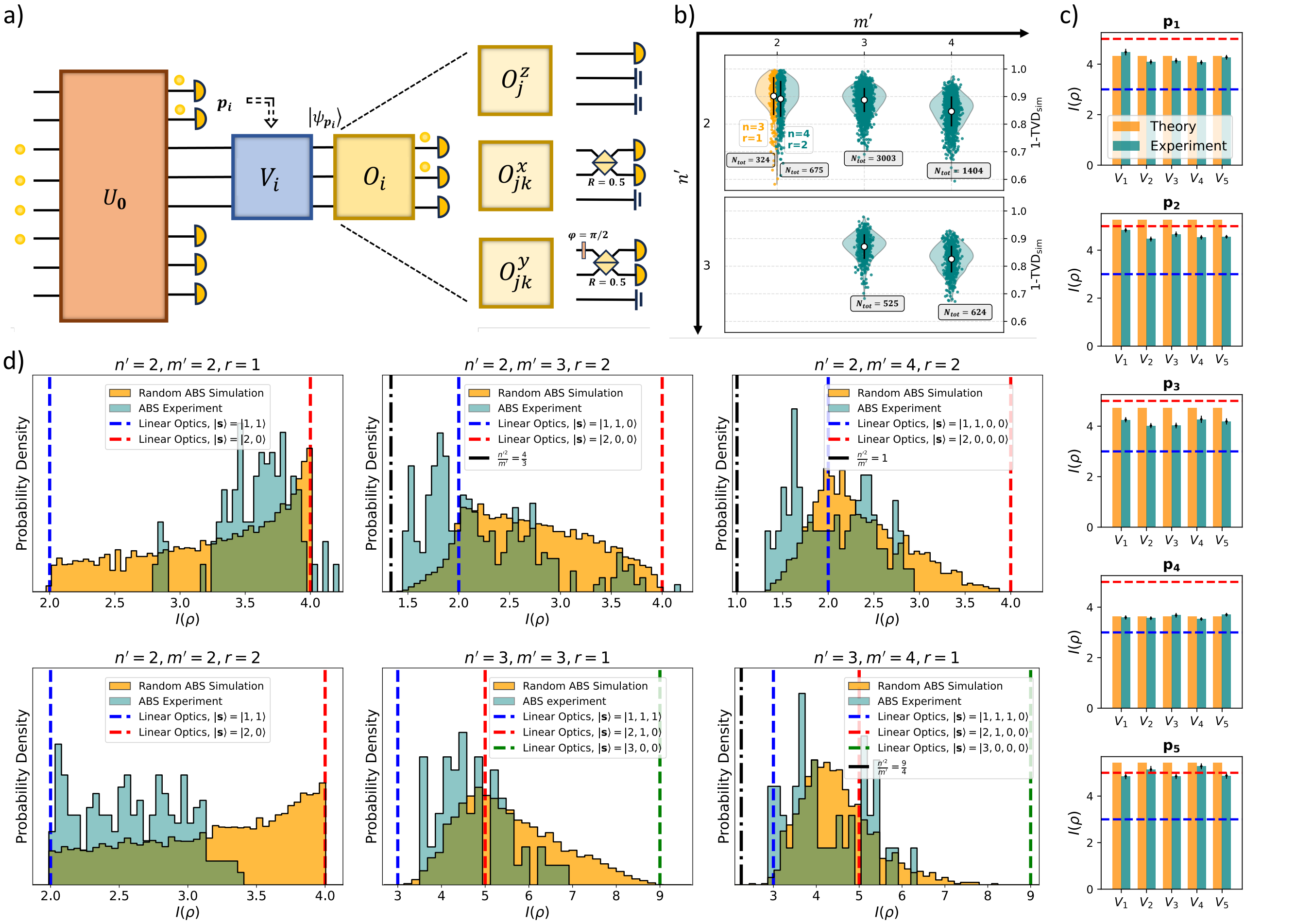


Supplementary Figure 5. **Details on the single-stage Adaptive Boson Sampling experiments for measuring Lie invariant quantities in the post-selection regime.** a) ABS interferometer scheme for measuring Lie observables with $m = 8$ modes and $n = 4$ input photons, shown in the specific case of $m' = 3$ output modes. The inset shows the transformations that need to be implemented to measure the expectation values of specific $O_j^z$, $O_{jk}^x$ and $O_{jk}^y$ observables. b) Violin plots for values of $1 - \mathbf{TVD}_{\rm sim}$, where $\mathbf{TVD}_{\rm sim}$ is the total variation distance with respect to numerical simulations (including a model accounting for experimental imperfections), for different configurations with varying number of output photons ($n'$) and modes ($m'$). Each data point corresponds to a distinct experimentally sampled configuration, including different input unitaries $U_0$, adaptive unitaries $V_i$ (including all considered permutations of the adaptive map), and measured observables $\hat{O}_i$. The total number of sampled configurations included in each violin plot is indicated as $N_{\rm tot}$. Violin plots represent the full distribution of sampled values. Central markers and error bars indicate mean values ± standard deviation. c) Measured values of $I(\hat{\rho})$ for a specific choice of $U_0$ in the $m' = 3$, $n' = 3$ configuration, compared with their ideal theoretical values. The results highlight the nonlinearity introduced by the measurement operation, which depends on the specific outcome $\boldsymbol{p}_i$. Each plot shows the values obtained when a different measurement outcome $\boldsymbol{p}_i$ is post-selected, with varying adaptive unitary $V_i$. Importantly, output states corresponding to the same $\boldsymbol{p}_i$ but different associated unitaries $V_i$ remain identical, since the evolution induced by $V_i$ is linear. Data are presented as mean values ± standard deviation, obtained from Monte Carlo resampling of the experimentally measured photon-count distributions assuming Poissonian counting statistics. The reported values of $I(\hat{\rho})$ are reconstructed from $N = 100$ resampled datasets. d) Histograms of Lie invariant quantities $I(\hat{\rho})$ obtained from single-stage ABS experiments. The two panels in the first column display histograms for $I(\hat{\rho})$ as obtained in the configurations with $n' = 2$ and $m' = 2$, for $r = 1$ and $r = 2$ measured photons, respectively. In particular, we probe three different $U_0$ unitaries for $r = 1$ and one $U_0$ for $r = 2$. Other panels display the histograms for measured $I(\hat{\rho})$ in scenarios with $m' = \{3, 4\}$ and $n' = \{2, 3\}$, with each experiment conducted for a set of three different $U_0$ unitaries. The histograms include results from different output states generated by considering permutations of the adaptive map. Vertical dashed lines indicate the theoretical values for $I(\hat{\rho})$ from Fock states with corresponding number of photons and modes, as well as the theoretical minimum and maximum values allowed in each configuration. In the plots we show also results obtained from simulation of random ABS output states within the corresponding $(m, n, k, r)$ configuration.

values of $\Delta_{\mathbf{TVD}} = 1 - \mathbf{TVD}_{\rm sim}$ obtained across the different $(n', m')$ configurations. The reported values are computed

over all initial unitaries $U_0$, adaptive unitaries $V_i$, including all considered permutations of the adaptive map, and measured observables $\hat{O}_i$, yielding average values of $\Delta_{\mathbf{TVD}}$ exceeding 0.8 in all configurations.

Finally, we emphasize that the nonlinearity affecting the invariant $I(\hat{\rho})$ originates from the measurement operation itself, which is emulated experimentally via post-selection. As a consequence, different values of $I(\hat{\rho})$ are observed for different measurement outcomes $\boldsymbol{p}_i$. However, for a fixed outcome $\boldsymbol{p}_i$, output states obtained after different choices of $V_i$ remain identical, since the evolution governed by each $V_i$ is purely linear. This behavior is explicitly illustrated in Supplementary Fig. 5-(c) for a representative choice of $U_0$ in the $m' = 3$, $n' = 3$ configuration.

### B. Two-stage ABS Experiments

We provide here additional details on the cascaded ABS protocol involving two intermediate measurements and two adaptive unitaries, as presented in the main text. This extended configuration allows us to explicitly investigate the role of adaptivity when multiple measurement-conditioned operations are applied sequentially, and to assess their impact on the Lie invariant $I(\hat{\rho})$.

The experiment is implemented with $n = 4$ input photons distributed over $m = 8$ modes. The photonic circuit is realized on a reconfigurable interferometric chip with a total of $m = 12$ modes, where four modes remain unused to provide the necessary interferometric depth and layout flexibility for the sequential stages. The overall evolution consists of a cascade of linear-optical transformations and intermediate measurements, as schematically illustrated in Supplementary Fig. 6-(a). The input state first evolves under an initial interferometer $U_0$ acting on eight modes. A first intermediate measurement is then performed on a subset of $k_1 = 4$ modes, detecting a single photon ($r_1 = 1$) and yielding a measurement outcome $\boldsymbol{p}^{(1)}$. This outcome conditions the application of a first adaptive unitary $V^{(1)}$, which acts on the remaining four modes. Subsequently, a second intermediate measurement is performed on two of the modes affected by $V^{(1)}$ ($k_2 = 2$), again detecting a single photon ($r_2 = 1$) and producing a measurement outcome $\boldsymbol{p}^{(2)}$. This second outcome conditions the application of a further adaptive unitary $V^{(2)}$, acting on the final two modes of the system. After this second adaptive step, the resulting output state contains $n' = 2$ photons in $m' = 2$ modes.

The final two-mode state is analyzed again by applying the additional linear-optical transformations that enable the measurement of the expectation values of the Lie observables $\hat{O}_i$, which are required to reconstruct the invariant $I(\hat{\rho}_{\mathrm{ABS}})$.

As in the single-stage ABS experiments discussed above, the protocol is implemented experimentally in a post-selection regime. This allows us to reinterpret the collected data under different associations between measurement outcomes and adaptive unitaries, effectively emulating different adaptive maps without active feed-forward. In particular, for fixed measurement patterns $(\boldsymbol{p}_i^{(1)}, \boldsymbol{p}_j^{(2)})$, we consider multiple choices of adaptive unitaries $V_i^{(1)}$ and $V_j^{(2)}$. Supplementary Fig. 6-(c) shows the experimentally obtained distribution of $I(\hat{\rho}_{\mathrm{ABS}})$ in this configuration, over two different choices of the initial unitary $U_0$.

To benchmark the reliability of the implementation, in Supplementary Fig. 6-(b) we report the distribution of $\Delta_{\mathbf{TVD}} = 1 - \mathbf{TVD}_{\mathrm{sim}}$, where $\mathbf{TVD}_{\mathrm{sim}}$ denotes the total variation distance with respect to numerical simulations including experimental imperfections. The distribution is evaluated across all measurement patterns $(\boldsymbol{p}_i^{(1)}, \boldsymbol{p}_j^{(2)})$, all choices of adaptive unitaries $(V_i^{(1)}, V_j^{(2)})$, including all considered permutations of the adaptive map, and all measured observables $\hat{O}_i$, yielding an average value of $\Delta_{\mathbf{TVD}} \sim 0.89$.

The experimentally obtained values of the Lie invariant $I(\hat{\rho}_{\mathrm{ABS}})$ are shown in Supplementary Fig. 6-(d) for a fixed choice of the initial interferometer $U_0$ and for different measurement patterns $(\boldsymbol{p}_i^{(1)}, \boldsymbol{p}_j^{(2)})$. For each measurement pattern, the invariant is reported as a function of the intermediate adaptive unitary $V^{(1)}$, and for two different choices of the final adaptive unitary $V^{(2)}$, and compared with the corresponding ideal theoretical values. Notably, while the value of $I(\hat{\rho}_{\mathrm{ABS}})$ is independent of the choice of $V^{(2)}$, which only induces a linear optical transformation applied after the final measurement, it depends not only on the measurement patterns $(\boldsymbol{p}_i^{(1)}, \boldsymbol{p}_j^{(2)})$, but also on the intermediate adaptive unitary $V^{(1)}$, since it is applied before the last measurement. We stress that, also in this two-stage configuration, the adaptive unitaries $V^{(1)}$ and $V^{(2)}$ were chosen according to a simple deterministic rule, without any optimization over the invariant values or other figures of merit, to avoid introducing any bias in the observed non-linear behavior.

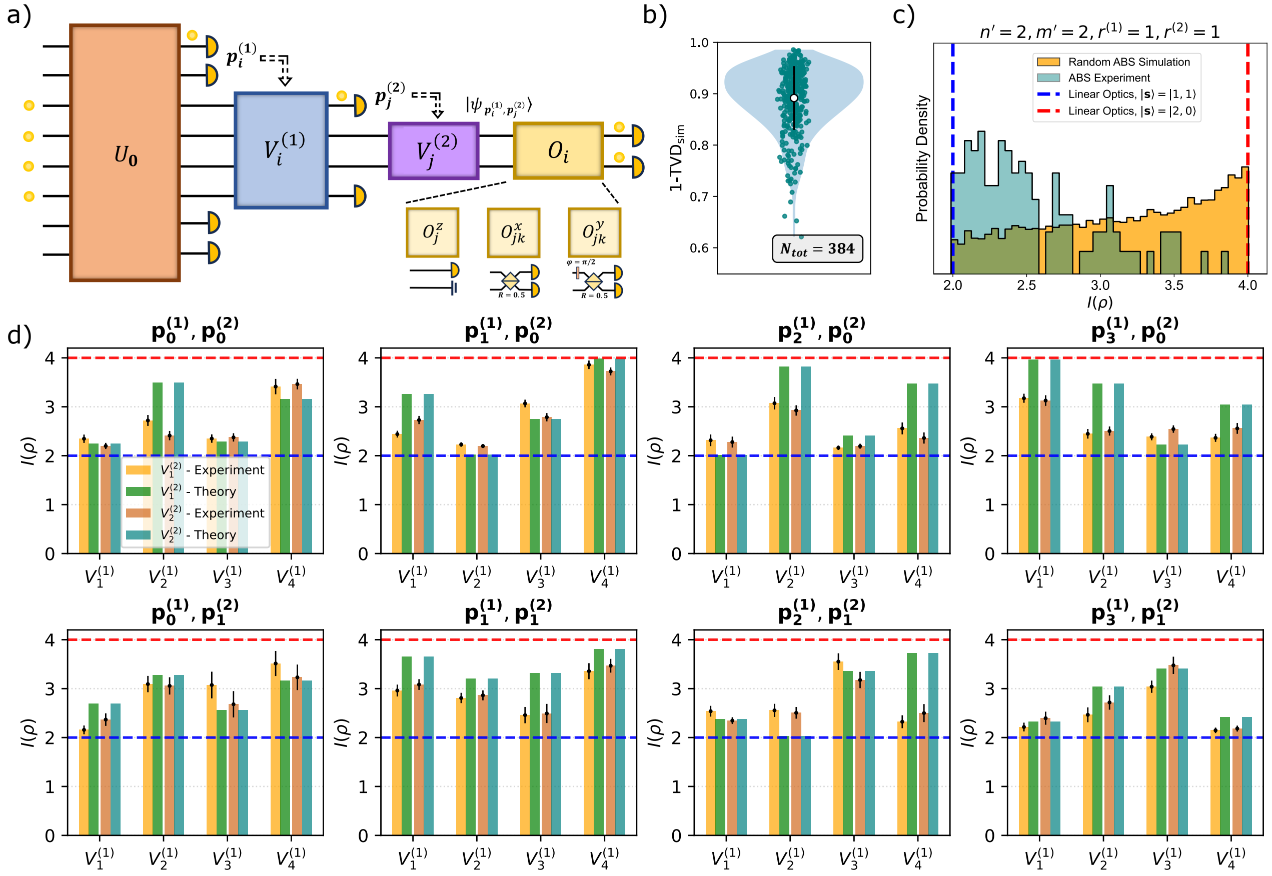


Supplementary Figure 6. **Details on the two-stage Adaptive Boson Sampling experiments for measuring Lie invariant quantities.** a) Schematic of the ABS with two adaptive measurements in cascade. Four photons are injected into 8 modes and evolve under an initial interferometer $U_0$. A first intermediate measurement $\boldsymbol{p}^{(1)}$ on four modes conditions the application of an adaptive unitary $V^{(1)}$, followed by a second measurement $\boldsymbol{p}^{(2)}$ on two modes conditioning a second adaptive unitary $V^{(2)}$. A projective transformation is applied to the resulting two-mode output state to measure the Lie observables $\hat{O}_i$ necessary for the evaluation of the invariant $I(\hat{\rho})$. b) Violin plot of $1 - \mathrm{TVD_{sim}}$, where $\mathrm{TVD_{sim}}$ is the total variation distance with respect to numerical simulations including experimental imperfections, evaluated over the different measurement outcomes, adaptive unitaries, and measured observables. Each data point corresponds to a distinct experimentally sampled configuration. The total number of sampled configurations is indicated in the panel as $N_{\mathrm{tot}}$. The violin plot represents the full distribution of sampled values. Central markers and error bars indicate mean values ± standard deviation. c) Histograms of Lie invariant quantities $I(\hat{\rho})$ obtained from two-stage ABS experiments. The histograms include results obtained probing two different $U_0$ unitaries and considering output states generated by permuting the adaptive map of the unitaries $V_i^{(1)}$ and $V_i^{(2)}$ to the measurement outcomes $(\boldsymbol{p_i^{(1)}}, \boldsymbol{p_j^{(2)}})$. Vertical dashed lines indicate the theoretical values for $I(\hat{\rho})$ from Fock states with two photons and two modes. In the plots we show also results obtained from simulation of random ABS output states within this configuration. d) Measured values of the invariant $I(\hat{\rho})$ for a fixed choice of $U_0$, shown for different measurement patterns $(\boldsymbol{p}_i^{(1)}, \boldsymbol{p}_j^{(2)})$. For each panel, the invariant is reported as a function of the adaptive unitary $V^{(1)}$, and for two different choices of $V^{(2)}$, compared with their ideal theoretical values. The results show that while the value of $I(\hat{\rho})$ is independent of $V^{(2)}$, which only induces a linear optical transformation after the last measurement, it depends on the measurement patterns but also on the adaptive unitary $V^{(1)}$, confirming that adaptivity contributes to the non-linear dynamics when implemented between sequential measurement stages. Data are presented as mean values ± standard deviation, obtained from Monte Carlo resampling of the experimentally measured photon-count distributions assuming Poissonian counting statistics. The reported values of $I(\hat{\rho})$ are reconstructed from $N = 100$ resampled datasets.

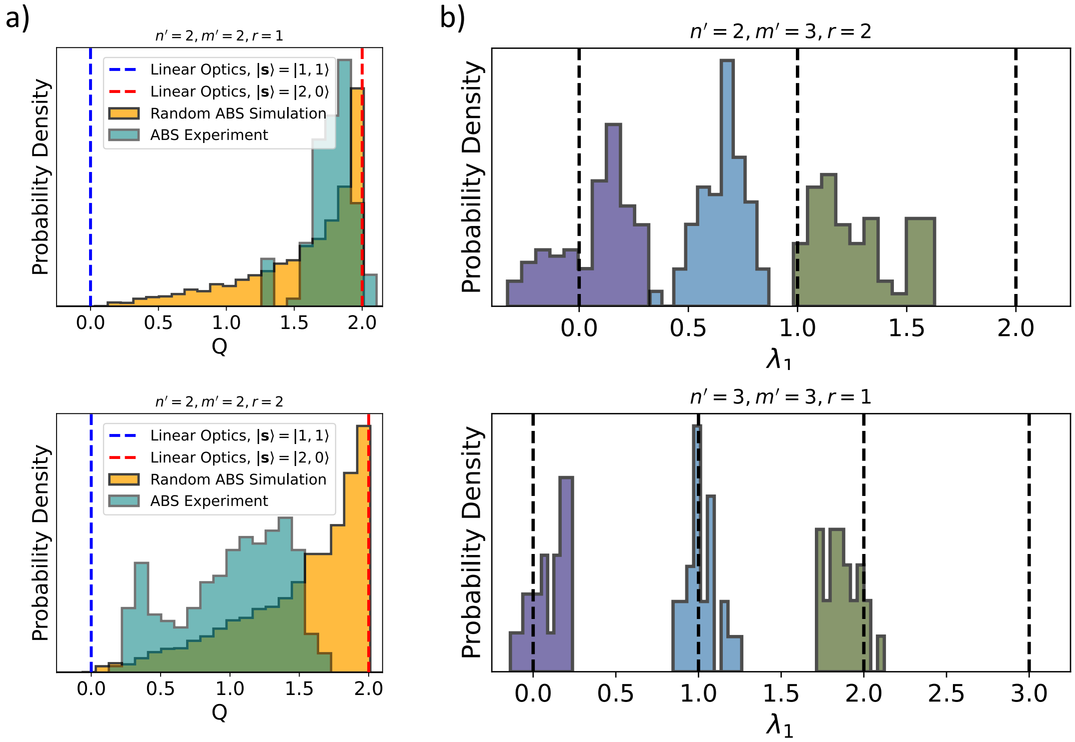


Supplementary Figure 7. **Witnessing nonlinearity in Adaptive Boson Sampling from spectral properties of $\hat{\rho}_T$.** a) Histograms of the measured values of the quantity $Q$, which fully determines the spectrum of $\hat{\rho}_T$ in the cases with $n' = 2$, $m' = 2$ and varying $r = \{1, 2\}$. Values obtained from post-selection single-stage ABS experiments are shown, together with simulated values from random ABS output states and the discrete set of values allowed from evolution of Fock state inputs thorough linear optics (indicated by vertical dashed lines). The continuous distribution of $Q$ highlights the emergence of non-linear dynamics, incompatible with purely linear-optical transformations. b) Histogram of the measured values for the eigenvalues $\{\lambda_1\}$ of the coherency matrix $\mathbf{\Gamma}^{(1)}$, corresponding to the spectrum of the first block of $\hat{\rho}_T$, in post-selection ABS experiments with $m' = 3$ and $n' = \{2, 3\}$. Here, dashed vertical lines indicate the discrete values obtainable via linear optics from Fock inputs, while the observed non-integer values demonstrate a clear departure from linear-optical constraints. Both panels showcase the ability of spectral quantities derived from $\hat{\rho}_T$ to witness non-linear behavior induced by the evolution in ABS.

## Supplementary Note 7. Witnessing non-linear dynamics from the spectrum of the Projection of the Density Matrix onto the Subalgebra of Linear Optical Hamiltonians

Given an output quantum state $\hat{\rho}$, it is possible to define the operator $\hat{\rho}_T$ as its projection onto the subalgebra generated by linear-optical observables $\{\hat{O}_i\}$. This is given by:

$$\hat{\rho}_T := \sum_i \mathrm{Tr}(\hat{O}_i \hat{\rho})\, \hat{O}_i. \tag{19}$$

In [7] it was shown that the spectrum of the operator $\hat{\rho}_T$ is invariant under linear optical evolution. This was also experimentally tested in [8] for BS experiments with different Fock State inputs of $m = \{2, 3\}$ modes.

Since all the operators $\hat{O}_i$ commute with the total photon number operator $\hat{N} = \sum_j \hat{a}_j^\dagger \hat{a}_j$, it follows that $\hat{\rho}_T$ is block-diagonal with respect to the total photon number, and each block acts on the $n$-photon subspace. Moreover, as already discussed in [8], the first block of $\hat{\rho}_T$, which corresponds to the $n = 1$ subspace, is structurally equivalent to the first-order coherency matrix $\mathbf{\Gamma}^{(1)}$, defined as

$$\mathbf{\Gamma}^{(1)}_{jk} := \langle \hat{a}_j^\dagger \hat{a}_k \rangle. \tag{20}$$

This matrix encodes the single-particle coherences between output modes.

In the following, the spectrum of $\hat{\rho}_T$ is experimentally examined under configurations that are equivalent to those analyzed in [8].

- $\boldsymbol{m' = 2}$: In this case, as shown in [8], the entire spectrum of $\hat{\rho}_T$ depends on a single scalar quantity $Q$:

$$\lambda_n^j = \frac{n}{2}(N_1 + N_2) + jQ, \qquad j \in \{-\frac{n}{2}, -\frac{n}{2}+1, \ldots, \frac{n}{2}-1, \frac{n}{2}\}, \qquad Q := \sqrt{(N_1 - N_2)^2 + 4|R_{12}|^2}, \tag{21}$$

where $N_j = \langle \hat{a}_j^\dagger \hat{a}_j \rangle$ and $R_{12} = \langle \hat{a}_2^\dagger \hat{a}_1 \rangle$. The quantity $Q$ measures the norm of the traceless part of $\Gamma^{(1)}$:

$$Q^2 = 2 \left\| \boldsymbol{\Gamma}^{(1)} - \frac{1}{2}\mathrm{Tr}[\boldsymbol{\Gamma}^{(1)}]\mathbb{I} \right\|^2, \tag{22}$$

and it is directly related to the Lie invariant $I(\hat{\rho}) = \sum_i \left(\mathrm{Tr}(\hat{O}_i\hat{\rho})\right)^2$ via the relation:

$$Q^2 = 2I(\hat{\rho}) - (N_1 + N_2)^2. \tag{23}$$

In Supplementary Fig. 7-(a) histograms for the values of $Q$ obtained in $m' = 2$, $n' = 2$ single stage ABS setups are reported, as evaluated from the measured values of $\mathrm{Tr}(\hat{O}_i\hat{\rho})$ in post-selected experiments. In linear optics, Fock inputs produce values of $Q$ that are quantized (in particular one has $Q = 0$ for states evolved from $|1,1\rangle$ input, and $Q = 2$ for states evolved from $|2,0\rangle$ input. In contrast, our experimental results for ABS show non-integer values of $Q$, revealing that the output state lies outside the set of states accessible via linear optics only.

- $\boldsymbol{m' = 3}$: Here, the first-order coherency matrix $\boldsymbol{\Gamma}^{(1)}$ is a $3 \times 3$ Hermitian matrix

$$\boldsymbol{\Gamma}^{(1)} = \begin{pmatrix} N_1 & R_{12} & R_{13} \\ R_{12}^* & N_2 & R_{23} \\ R_{13}^* & R_{23}^* & N_3 \end{pmatrix}, \tag{24}$$

where $N_j = \langle \hat{a}_j^\dagger \hat{a}_j \rangle$ and $R_{jk} = \langle \hat{a}_k^\dagger \hat{a}_j \rangle$. As shown in [8], the eigenvalues $\{\lambda_1\}$ of $\boldsymbol{\Gamma}^{(1)}$ coincide with those of the first block of $\hat{\rho}_T$. In Supplementary Fig. 7-(b) histograms for the values of $\{\lambda_1\}$ as obtained in $m' = 3$ ABS setups are reported. These values are obtained by diagonalizing numerically the matrix in Eq. (24), whose entries are obtained from the measured values of $\mathrm{Tr}(\hat{O}_i\hat{\rho})$ in post-selected experiments. Under linear-optical evolution from Fock inputs, these eigenvalues are constrained to be integers, corresponding to the occupation numbers $n_j$ of the input state. (e.g., evolving $|2,0,1\rangle$ input one gets $\{\lambda_1\}$ =$\{0,1,2\}$ ). However, in our ABS experiments, we observe non-integer eigenvalues, demonstrating that the output states are incompatible with evolution under linear optics alone.

These results confirm that the spectral properties of $\hat{\rho}_T$ and $\boldsymbol{\Gamma}^{(1)}$ provide an alternative practical witness of non-linearity in ABS.

## Supplementary Note 8. Measuring Lie invariant quantities for mixed ABS outputs

In the ABS protocol, the output state of the system depends on the outcomes of intermediate measurements. In our experiments, we fix the number of measured photons $r$ and the number of measurement modes $k$. This leads to a constant number of possible adaptive outcomes for each experiment.

While in the main text we considered the output state $|\psi_{\boldsymbol{p}}\rangle$ for each post-selected outcome $\boldsymbol{p}$, here we aim to characterize the structure of an unconditioned ABS output by reconstructing the Lie algebraic invariants associated with the effective mixed state obtained by averaging over all post-selected outcomes.

In the following, we restrict the analysis to single-stage ABS protocols. Moreover, we consider only data from experiments where adaptivity is emulated via post-selection, allowing us once again to consider different permutations of the adaptive map. For each configuration $(n', m')$, we consider all adaptive outcomes corresponding to the different considered combinations, each with a fixed number $r$ of measured photons. Each adaptive evolution, described by the pair $(\boldsymbol{p}_i, V_i)$, yields a conditional output state $|\psi_{\boldsymbol{p}_i}\rangle$, evolved under a unitary $V_i$ after the measurement outcome $\boldsymbol{p}_i$ is observed. We then form the mixed state

$$\hat{\rho} = \sum_{i=1}^{M} p(\boldsymbol{p}_i) \, |\psi_{\boldsymbol{p}_i}\rangle \langle\psi_{\boldsymbol{p}_i}| \, , \tag{25}$$

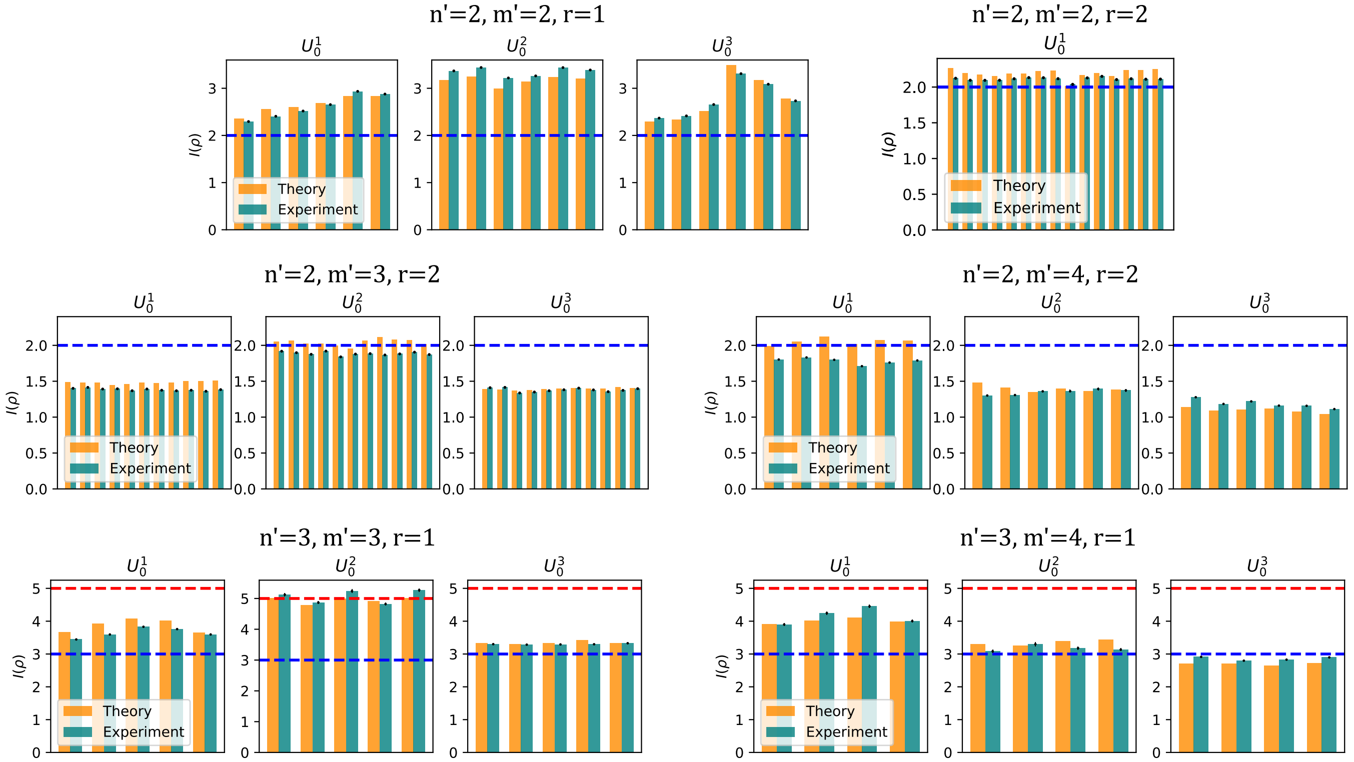


Supplementary Figure 8. **Lie invariants $I(\hat{\rho})$ reconstructed from mixed states in Adaptive Boson Sampling (ABS).** All data shown here correspond to experiments in the post-selection regime. Top panels show the values of $I(\hat{\rho})$ in the $m' = m - k = 2$, $n' = n - r = 2$ configuration, for both $r = 1$ and $r = 2$. Bottom panels display the values of $I(\hat{\rho})$ in experiments with varying configurations $m' = \{3, 4\}$ and $n' = \{2, 3\}$. In each configuration, the analysis is repeated for the distinct instances of the initial interferometer $U_0$, and each subplot corresponds to one such instance. For all experiments, the invariant is reconstructed from the mixed state obtained by averaging over $M$ cyclic permutations of uniquely associated adaptive measurement outcomes and corresponding unitaries. Data are presented as mean values ± standard deviation, obtained from Monte Carlo resampling of the experimentally measured photon-count distributions assuming Poissonian counting statistics. The reported values of $I(\hat{\rho})$ are reconstructed from $N = 100$ resampled datasets. In several cases, the error bars are smaller than the marker size and are therefore not visible. Horizontal dashed lines correspond to the values of $I(\hat{\rho})$ for Fock states with the corresponding number of photons and modes.

where $M$ is the number of distinct $(\boldsymbol{p}_i, V_i)$ pairs and $p(\boldsymbol{p}_i)$ is the probability of obtaining the outcome $\boldsymbol{p}_i$, normalized over all the considered outcomes. This state captures the effective output of the ABS protocol when one does not condition on a specific adaptive outcome, but instead samples from the whole ensemble.

To ensure an unbiased sampling over possible associations between adaptive outcomes and unitaries, we perform $M$ cyclic permutations of the $(\boldsymbol{p}_i, V_i)$ pairs, following the same strategy of the kernel-based analysis of adaptive maps discussed in Supplementary Note 4:

$$(\boldsymbol{p}_1, V_1), (\boldsymbol{p}_2, V_2), \ldots, (\boldsymbol{p}_M, V_M) \to (\boldsymbol{p}_1, V_2), (\boldsymbol{p}_2, V_3), \ldots, (\boldsymbol{p}_M, V_1) \to \quad \cdots \tag{26}$$

Each such permutation defines a different way to pair the adaptive outcomes $\boldsymbol{p}_i$ with unitaries $V_i$, resulting in a new mixed state $\hat{\rho}$ formed from the corresponding conditional pure states. This procedure avoids redundancy while systematically exploring the space of possible adaptive maps, allowing us to construct $M$ distinct density matrices per experiment.

For each of the $M$ mixed states, the associated Lie invariant is evaluated using the method presented in the main text by applying the weighted averaging of Eq. (25) to the output probability distributions obtained for each Lie observable. The weighted averaging is performed across all $M$ adaptive configurations $(\boldsymbol{p}_i, V_i)$ within a given permutation.

In all cases, since experimental acquisition rates vary across adaptive configurations, we avoid biasing the ensemble average by estimating the weights $p(\boldsymbol{p}_i)$ from numerical simulations of the ideal protocol. This yields a rate-independent mixed state that faithfully reflects the ideal scenario. The analysis is repeated for each of the $M$ cyclic permutations.

Each experiment is repeated using all available, independently sampled initial unitaries $U_0$. In Supplementary Fig. 8, for each $(n', m')$ configuration, subplots corresponding to the different $U_0$ instances are presented. For each subplot, the values for the Lie invariant $I(\hat{\rho})$ corresponding to the mixed state obtained from the $M$ cyclic permutations are displayed. Notably, non-linear features are evident also in the Lie invariant quantities associated with these mixed states, as values differing from those of Fock states are obtained. Furthermore, while the non-conservation of $I(\hat{\rho})$ is already evident at the level of pure output states and arises solely from the measurement process, the fact that different cyclic permutations yield different values of $I(\hat{\rho})$ reflects a genuine feature of the adaptive evolution. This effect stems from the influence of the varying unitaries $V_i$ applied after each measurement outcome, which cannot be factored out in the evaluation of the Lie invariant $I(\hat{\rho})$.

To make this point explicit, consider the Lie invariant evaluated on a mixed state:

$$I(\hat{\rho}) = \sum_i \left(\mathrm{Tr}(\hat{O}_i\hat{\rho})\right)^2, \tag{27}$$

where the output state is the mixed state given by

$$\hat{\rho} = \sum_j p(\boldsymbol{p}_j)\hat{V}_j \, |\phi_{\boldsymbol{p}_j}\rangle \langle\phi_{\boldsymbol{p}_j}| \, \hat{V}_j^\dagger. \tag{28}$$

Here, we explicitly denoted with $|\phi_{\boldsymbol{p}_j}\rangle$ the pure state resulting from just tracing out a subset of modes (conditioned on obtaining a given outcome $\boldsymbol{p}_j$) and with $\hat{V}_j$ the unitary operator describing its evolution.

While each individual state $|\phi_{\boldsymbol{p}_j}\rangle$ and its evolved counterpart $\hat{V}_j |\phi_{\boldsymbol{p}_j}\rangle$ yield the same value of the invariant, i.e.,

$$I(\hat{V}_j |\phi_{\boldsymbol{p}_j}\rangle \langle\phi_{\boldsymbol{p}_j}| \, \hat{V}_j^\dagger) = I(|\phi_{\boldsymbol{p}_j}\rangle \langle\phi_{\boldsymbol{p}_j}|), \tag{29}$$

this equivalence does not extend to the mixed state $\hat{\rho}$. Specifically,

$$I(\hat{\rho}) = \sum_i \left[\mathrm{Tr}\left(\hat{O}_i \sum_j p(\boldsymbol{p}_j)\hat{V}_j |\phi_{\boldsymbol{p}_j}\rangle \langle\phi_{\boldsymbol{p}_j}| \hat{V}_j^\dagger\right)\right]^2 \neq \sum_j p(\boldsymbol{p}_j) I(|\phi_{\boldsymbol{p}_j}\rangle \langle\phi_{\boldsymbol{p}_j}|). \tag{30}$$

The reason is twofold. First, as shown in [8], the individual expectation values $\mathrm{Tr}(\hat{O}_i\hat{\rho})$ are not themselves invariant under a linear evolution of $\hat{\rho}$; only the full expression $I(\hat{\rho})$ is. Second, and more importantly in this context, $I(\hat{\rho})$ is a non-linear functional of $\hat{\rho}$ due to the presence of the square over the sum of expectation values. As a result, even if each pure state yields the same value for the invariant, their convex combination can lead to a different one, since squaring and averaging do not commute. In this way, the square renders the invariant sensitive to the statistical mixing introduced by the adaptive scheme, effectively revealing the impact of outcome-dependent unitaries. The observed variation of $I(\hat{\rho})$ across cyclic permutations is thus a direct signature of the adaptive nature of the evolution. This observation further highlights that the choice of the outcome-dependent unitaries $\hat{V}_j$ directly affects the structure of the effective mixed output state. While in the present work the adaptive map is fixed according to a simple deterministic prescription and is not optimized for any specific task, in general one could engineer the mapping $(\boldsymbol{p}_j, V_j)$ to tailor properties of the mixed state $\hat{\rho}$, for instance enhancing its purity or other desired figures of merit.

## Supplementary References

[1] R. T. Thew, K. Nemoto, A. G. White, and W. J. Munro, Physical Review A **66**, 012303 (2002).
[2] G. Kimura, Physics Letters A **314**, 339–349 (2003).
[3] H. Hurwitz and R. C. Jones, Journal of the Optical Society of America **31**, 493 (1941).
[4] J. Řeháček, Z. Hradil, E. Knill, and A. I. Lvovsky, Physical Review A **75**, 042108 (2007).
[5] M. C. Tichy, Physical Review A **91**, 022316 (2015).
[6] M. Pont, R. Albiero, S. E. Thomas, N. Spagnolo, F. Ceccarelli, G. Corrielli, A. Brieussel, N. Somaschi, H. Huet, A. Harouri, A. Lemaître, I. Sagnes, N. Belabas, F. Sciarrino, R. Osellame, P. Senellart, and A. Crespi, Physical Review X **12**, 031033 (2022).
[7] P. V. Parellada, V. G. i Garcia, J. J. Moyano-Fernández, and J. C. Garcia-Escartin, "Lie algebraic invariants in quantum linear optics," (2024), arXiv:2409.12223 [quant-ph].
[8] G. Rodari, T. Francalanci, E. Caruccio, F. Hoch, G. Carvacho, T. Giordani, N. Spagnolo, R. Albiero, N. Di Giano, F. Ceccarelli, G. Corrielli, A. Crespi, R. Osellame, U. Chabaud, and F. Sciarrino, Physical Review Research **7**, 043325 (2025).
